# Morbidity and mortality among children, adolescents, and young adults with cancer over six decades: a Swedish population-based cohort study (the Rebuc study)

**DOI:** 10.1016/j.lanepe.2024.100925

**Published:** 2024-05-15

**Authors:** Margaretha Stenmarker, Panagiotis Mallios, Elham Hedayati, Kenny A. Rodriguez-Wallberg, Aina Johnsson, Joakim Alfredsson, Bertil Ekman, Karin Garming Legert, Maria Borland, Johan Mellergård, Moa Eriksson, Ina Marteinsdottir, Thomas Davidson, Lars Engerström, Malte Sandsveden, Robin Keskisärkkä, Martin Singull, Laila Hubbert

**Affiliations:** a Department of Oncology, Department of Biomedical Sciences and Clinical Sciences, Linkoping University, Linkoping, Sweden; b Department of Cardiology and Department of Health, Medicine and Caring Sciences, Linkoping University, Norrkoping, Sweden; c Department of Oncology-Pathology, Karolinska Institutet, Stockholm, Sweden; d Department of Cardiology and Department of Health, Medicine and Caring Sciences, Linkoping University, Linkoping, Sweden; e Department of Endocrinology in Linkoping, Department of Internal Medicine in Norrkoping, and Department of Health, Medicine and Caring Sciences, Linkoping University, Linkoping, Sweden; f Department of Dental Medicine, Karolinska Institutet, Stockholm, Sweden; g Institute of Neuroscience and Physiology, Department of Health and Rehabilitation/Physiotherapy, Sahlgrenska Academy, University of Gothenburg, SV Hospital Group Rehabilitation Centre, Alingsas Hospital, Alingsas, Sweden; h Department of Neurology in Linkoping, and Department of Biomedical and Clinical Sciences, Linkoping University, Linkoping, Sweden; i Department of Ophthalmology and Department of Biomedical and Clinical Sciences, Linkoping University, Linkoping, Sweden; j Department of Medicine and Optometry, Faculty of Health and Life Sciences, Linnaeus University, Kalmar, Sweden; k Department of Health, Medicine and Caring Sciences, Linkoping University, Linkoping, Sweden; l Department of Anesthesia and Intensive Care in Norrkoping and Department of Medical and Health Sciences, Linkoping University, Sweden; m Department of Surgery and Department of Biomedical and Clinical Sciences, Linkoping University Norrkoping, Sweden; n Department of Computer and Information Science, Linkoping University, Linkoping, Sweden; o Department of Mathematics, Linkoping University, Linkoping, Sweden; p Department of Paediatrics, Institute of Clinical Sciences, Sahlgrenska Academy, University of Gothenburg, Gothenburg, Sweden; q Department of Paediatrics, Futurum Academy for Health and Care, Region Jonkoping County, Sweden; r Medical Unit: Breast, Endocrine Tumours, and Sarcoma, Theme Cancer, Karolinska University Hospital and Karolinska Comprehensive Cancer Centre, Stockholm, Sweden; s Department of Reproductive Medicine, Division of Gynecology and Reproduction, Karolinska University Hospital, Stockholm, Sweden

**Keywords:** Childhood cancer, CAYA, Socio-economic, Demographics, Survival, Treatment side effects

## Abstract

**Background:**

Despite progress in managing cancer in children, adolescents, and young adults (CAYAs), persistent complications may impact their quality of life. This study covers the morbidity and mortality, among CAYAs, with the aim to investigate the influence of socioeconomic factors on outcomes.

**Methods:**

This retrospective matched cohort study included the entire Swedish population of individuals under 25 with cancer 1958–2021. The population was identified from the Cancer Register, and controls were paired 1:5 based on age, sex, and residence. Multiple registers provided data on morbidity, mortality, and demographics.

**Findings:**

This survey covering 63 years, identified 65,173 CAYAs and matched controls, a total of 378,108 individuals (74% females). CAYAs exhibited a 3.04-times higher risk for subsequent cancer (Odds ratio (OR) 95% confidence interval (CI) 2.92–3.17, p < 0.0001), a 1.23-times higher risk for cardiovascular disease (OR 95% CI 1.20–1.26, p < 0.0001), and a 1.41-times higher risk for external affliction (OR 95% CI 1.34–1.49, p < 0.0001). CAYAs had a higher mortality hazard, and after adjusting for socioeconomic factors, males, individuals born outside Europe, and those with greater sick-leave had a higher association with mortality, while education and marriage showed a beneficial association.

**Interpretation:**

The Rebuc study, showed an increased risk for serious complications among young cancer patients in Sweden. Patient-specific variables, demographics, and socioeconomic factors influenced mortality. These results underscore the impact of cancer on the health and lifespan of young individuals and the necessity for further research to address socioeconomic disparities in cancer care.

**Funding:**

Grants from the 10.13039/100010805Medical Research Council of Southeast Sweden (FORSS), ALF Grants, Region Ostergotland, and The Swedish Childhood Cancer Fund.

**Research in context**

**Evidence before this study**

Previous large cohort studies on young cancer survivors have extensively documented long-term morbidity and mortality across diverse age groups and populations. It is well-established that cancer treatment itself can lead to lifelong or life-threatening conditions in this population. Furthermore, differences across countries in living conditions, access to healthcare, and variations in healthcare systems may influence long-term outcomes. To identify relevant studies, we conducted a thorough search using terms such as cohort study, register study, childhood cancer, adolescence cancer, CAYA, AYA, long-term survivor, late mortality, late morbidity, cardiovascular toxicity, subsequent malignancy, secondary malignancy, socioeconomic disparity, and financial toxicity. A systematic search from 2000 to 2023 was performed in UniSearch, Linkoping University's library search tool, encompassing 154 licensed databases and a vast collection of printed and electronic material, including 747,434 papers/books, 454,007 e-books, and 21,180 e-journals. Furthermore, searches were conducted in PubMed and the Cochrane Library, and subsequent searches thereafter.

**Added value of this study**

This study significantly contributes to the existing literature in that it is the first comprehensive report that compares an entire population of young cancer patients in Sweden with matched controls. This approach adds a novel dimension to our understanding of what long-term outcomes and challenges young cancer survivors face. With a dataset of 378,108 individuals, including 65,173 child, adolescent, and young adults (CAYAs) matched with 312,935 controls over 63 years between 1958 and 2021, this retrospective register study fills a critical gap in our knowledge, laying the foundation for evidence-based healthcare policies.

Beyond quantification of risks, the study systematically investigates morbidity, mortality, and a spectrum of individual and demographic variables. This in-depth analysis sheds light on long-term socioeconomic disparities, enabling tailored interventions and support systems. Notably, this study is novel in that it also covers all non-cancerous diseases occurring within five years of being diagnosed with cancer, an aspect that is often left out in previous studies. This comprehensive approach enriches our understanding of the multifaceted health challenges facing CAYAs.

Furthermore, this study goes beyond childhood cancer by following patients up to 25 years of age enabled the inclusion of all cervical cancer (including locally treatable cancer detected at screening) and shedding light on a large group of young women with cancer whose long-term outcome has been poorly investigated. Inclusion of this previously underexplored group enhances the generalisability of our findings, catering for the wide spectrum of young cancer survivors.

**Implications of all the available evidence**

The findings of this study, together with our existing knowledge, will have crucial implications for healthcare practice and policy regarding young patients with cancer. Based on the risks identified, healthcare practitioners will now be able to tailor interventions to the needs of their young cancer survivors. Subsequent long-term care of cancer survivors is imperative considering the substantial reduction in life expectancy for many, and policymakers must allocate resources to improve the situation of these individuals. Comprehensive cancer registers must include all malignancies over time, and the data thus gathered used to guide healthcare planning for equitable post cancer care, particularly for those with socioeconomic disparity.

Furthermore, this study stresses the need to address the underexplored aspects of care of young women survivors, and we urge policymakers to incorporate these insights into the healthcare of these women. Future research should focus on causal relationships, with emphasis on long-term survival experiences of young adults. Evaluating the effectiveness of interventions is key to guiding evidence-based practice, improving the outcomes of young cancer survivors. Together, such efforts will contribute to a more nuanced understanding of the challenges faced by this population, providing informed strategies for comprehensive care tailored to the individual patient.

This study is novel in that it also covers all non-cancerous diseases that happen within five years of being diagnosed with cancer, an aspect that is often left out in previous studies.

## Introduction

Due to progress in research and medical interventions, cancer management in children, adolescents, and young adults (referred to as CAYAs) has achieved notable success. In Sweden, the five-year survival for children and adolescents with cancer is approximately 85%, with survival increasing to about 90% for young adults up to 25 years of age.^1^

Many national and international CAYAs survival cohorts have been set up in the past decades to study the health problems and mortality of young cancer patients.^2^ From these it has been reported that cancer survivors may face various long-term complications later in life, which may have a significant negative impact on their quality of life (QoL), and some of these can be severe or life-threatening. The primary nature of these conditions includes subsequent malignancy, cardiovascular disease (CVD), central nervous system (CNS) disease, endocrine disease, infertility, and others.^3^, ^4^, ^5^ Furthermore, physiologic weakness (frailty) and premature aging have been reported among survivals.^6^

It is well-known that treatment with radiotherapy (RT) and cytostatics such as anthracyclines are associated with a dose dependent increased risk for late morbidity among young patients with cancer.^7^^,^^8^ In addition to the harm of treatment itself, the future QoL and cancer outcomes also depend on several common risk factors. Smoking, age, sex, heredity, hypertension, alcohol overconsumption, and metabolic syndrome are known risk factors for both cancer and CVD.^9^^,^^10^ Female survivors are at higher risk for CVD, and this is related to treatment, age at treatment, and the presence of certain risk factors such as obesity, diabetes, and high blood pressure. However, male survivors show CVD earlier than female survivors, possibly because they are more likely to have hyperlipidaemia and hypertension, and smoke more.^11^ There is also a link between dental health and overall health, as dental infection can raise the chances of having diabetes, high blood pressure, negative pregnancy outcomes, and CVD.^12^

A Scandinavian study found a 10% overall mortality among young cancer survivors before reaching middle age.^4^ The mortality rates are comparable in large studies from Europe and America,^9^^,^^13^ but the mortality has decreased among survivors in recent decades.^14^

Healthcare inequality due to socioeconomic status is omnipresent and may have an impact on the outcome of cancer. Cancer survivors may suffer consequences due to an adverse financial situations and burdens to which the patients and their families are subjected. Financial embarrassment may have direct emotional, psychological, practical consequences as well as indirect consequences such as interruption of studies, reduced employability, and reduced lifetime income.^15^, ^16^, ^17^, ^18^, ^19^ The socioeconomic status of the child's environment also plays a role i.e., feeling of security, healthy living conditions, opportunity to exercise, and access to healthy foods important for physiologic reserve and avoidance of frailty after childhood cancer.^20^

Due to variations in general healthcare and demographics, also between the Nordic countries, it is imperative to consider local conditions when formulating risk prevention strategies. Sweden benefits from a relatively small population, and easy access to healthcare and socioeconomic data over time allows comprehensive follow-up of the cohort over many years, encompassing aspects such as survival, health, education, and socioeconomic factors.

By integrating register data on healthcare and socioeconomic factors with geographical information, valuable insights into the complications of cancer and its treatment can be obtained. This approach sheds light on healthcare structure, education, socioeconomic status, and geographic factors on the lives of young cancer patients. Such knowledge is crucial for shaping the future landscape of cancer treatment and care. Hence, we have developed a big national database called Rebuc (register study of long-term outcomes of child, adolescent, and young adult cancer in Sweden) that is intended for use in various cohort studies.

### Objectives

The main objectives of this study were to describe the morbidity and mortality of all young Swedish cancer patients over the last six decades and compare them with matched controls to study the patient-specific variables, demographic, and socioeconomic factors in patients with cancer at a young age.

The purpose of this study was to provide a comprehensive picture of cancer among young people, including its demographic variation. Additionally, we aim to examine the prevalence of subsequent cancer among five-year survivors, as well as all non-cancer diseases that develop within five years and beyond. We suggest that demographic and socioeconomic factors may influence all-cause mortality. There are no previous comprehensive register studies that cover these aspects.

## Methods

### Study design and participants

This was a register-based, matched cohort study on the total Swedish population of cancer patients below 25 years of age, from January 1958 to the time of mortality or end of study in December 2021.

The National Board of Health and Welfare's Cancer Register (from 1958) was used to identify the study population by retrieving data on cancer diagnoses and their corresponding dates (referred as index). All 65,173 CAYAs in the index group were paired 1:5, by the Statistics Authority, Statistics Sweden (SCB) (from 1960), with controls based on year of birth, sex, the same municipality of residence, and freedom from cancer at the time of index. This matching process resulted in 312,935 controls (Appendix p 2).

The Swedish Ethics Review Authority approved this study, and the work complied with Strengthening the Reporting of Observational Studies in Epidemiology (STROBE) guidelines and the Declaration of Helsinki. According to Swedish legislation, patients registered in a national healthcare quality register need not provide written informed consent for their data to be included in healthcare research or published. Pseudonymised data were extracted via the National Board of Health and Welfare.

### Procedures

Data on all 378,108 individuals were matched between several population and healthcare registers, using the personal identity number assigned to all Swedish residents. The personal identification numbers assigned at birth indicate the assigned sex by the penultimate. A new personal identification number provided during a gender change, is not automatically linked to the previous number.

Diagnosis information was collected using the WHO International Classification of Diseases ICD codes 7–10. (Appendix p 4) The National Patient Register (from 1964) was used to obtain in-hospital care time and diagnoses (registered at discharged), and other diagnoses were collected from the outpatient section (from 2001) of the same register. We included all registered primary, secondary, and tertiary diagnoses and recorded the first occurrence of each. Intensive care data were extracted from the Swedish Intensive Care Register, (SIR) (from 2001), and data on intact teeth remaining from the National Dental Health Register (from 2008). The Swedish Cause of Death register (from 1958) was used for time and cause of death.

Information on population characteristics, demographics, and socioeconomic factors such as; residence at time of index cancer, 2021 years median income including income inequality (Gini-coefficient) at place of residence, latest registered civil status (unmarried, married), sex (at index), highest education level, (completed elementary school, upper secondary school, university, or postgraduate education), numbers of days sick-leave (between the age of 18 and 66 years) and disability pension (for unemployed over the age of 19 years) were extracted from the longitudinal integrated database for health insurance and labour market studies (LISA) (from 1990), SCB (from 1960) and the National Insurance Agency (from 1990). The registers and the time span during which they were used are explained in Appendix pages 2 and 3.

The median income of the local municipality (2021) was used as a surrogate for income as well as the socioeconomic development of the place of residence over time. Previous municipality identification numbers were converted into numbers as of 2021 and used when calculating the driving distance to the nearest hospital using Google Maps API (Mountain View, CA, USA). Standard protocols for treatment since 1958 (obtained from pioneers in Swedish paediatric oncology, colleagues, and centres) were used as surrogates for treatment with RT, anthracyclines, and other drugs (others) for leukaemia, CNS malignancies, Hodgkin's lymphoma, non-Hodgkins's lymphoma, and testis cancer.

### Statistical analysis

The primary outcome measures were morbidity and mortality between index diagnosis date (referred to as index) and the end of follow-up. The secondary outcome was the link between socioeconomic status and mortality.

CAYAs were compared with matched controls regarding baseline characteristics and descriptive analyses, and with all the measures described below. CAYAs and controls characteristics and baseline data are summarised with descriptive statistics for the entire cohort (total) and for the different age groups (<1–14, 15–18 and 19–24 years). CAYAs still alive five years after index were referred to as five-years survivors. Due to changes in cancer therapy and medical healthcare in general over the six decades of this study, observations were also grouped into periods 1958–1970, 1971–1980, 1981–1990, 1991–2000, 2001–2010, and 2011–2021.

Categorical variables were reported as counts (n), proportions in percentages (%), and continuous variables with median and interquartile ranges (IQR) due to skew distributions. For categorical data, between-group differences were tested for statistical significance using two-sample tests of proportion with a normal approximation. For continuous variables between-group difference were tested using Wilcoxon rank-sum test or difference in group means using normal approximation based on the central limit theorem. In few cases, when the approximation is not valid, the test is omitted, and no p-values are reported. Analyses at age 30 years an older were only preformed regarding education and civil status for adjustment due to early morbidity and mortality, which may hinder social progression.

The odds ratio (OR), the measure of how strongly an event (morbidity) is associated with being a CAYAs, was derived. The OR is a ratio of two sets of odds: the odds of the event occurring in the CAYAs group vs the odds of the event occurring in the control group. The risk of having a subsequent or new primary malignancy as well as other diseases was analysed and adjusted OR (with a 95% confidence interval (CI)) between CAYAs and controls was examined and presented on a forest plot. Considering matching, the ORs were adjusted for the matched control variables, i.e., age, sex, and part of Sweden at index. Hence, we use logistic regression model including those variables and the additional specific variable of interest.

The various times for mortality was reported as median (IQR) while cumulative mortality was illustrated with Kaplan–Meier curves including sex and age groups. Kaplan–Meier curves was also used to illustrate all cause, cancer specific and cardiovascular mortality over the decades and for 12 index diagnosis.

Hazard Ratio (HR) with a 95% CI was reported for CAYAs and Controls for various factors and covariates. For the survival analysis Cox regression models were used with start January 1, 1958, origin as the index dates for the CAYAs and for respectively controls. The end times used are the time of mortality or end of study in December 2021. Univariable Cox regression analysis was performed to examine the socioeconomic factors on all-cause mortality. (predictors are listed in Appendix p 6). Additionally, multivariable Cox regression models was used to adjust for various socioeconomic factors on all-cause mortality. We used the enter method, i.e., entered all the variables in the multivariable model simultaneously. We used the age at index (years) for each individual as variable and not the three age groups and decade at index. Hence, the multivariable Cox regression model is adjusted for the matched control variables as we did for the ORs. Finally, the underlying assumptions for the Cox regression model were assessed studying different residuals, such as martingale and Schoenfeld residuals. No major model violations were found, and the models were accepted for the purposes of this manuscript.

#### Methods for handling missing data

When missing data for the socioeconomic factors (listed in Tables 1 and 2), the individuals are omitted in the Cox regression model analyses. Out of 19,703 cases, 5.2% of mortality entries were inaccurately recorded in the cause of death register. In some instances, the year and months of mortality were recorded, but the day was missing. The last day of the respective months was appended to facilitate the calculation of time to mortality.

**Table 1:** Baseline demographic and clinical and socioeconomic characteristics of 65,173 patients with index malignancy and their 312,935 matched controls.

Age group at index cancer	CAYAs n = 65,173				Controls n = 312,935			
	<1–14	15–18	19–24	Total	<1–14	15–18	19–24	Total
n (%)	15,293 (23.5)	6639 (10.2)	43,241 (66.3)	65,173	69,189 (22.1)	31,071 (9.9)	21,2675 (68.0)	312,935
Age at diagnosis, years median (IQR)	5 (2–10)	17 (16–18)	23 (21–24)	21 (15–23)	5 (2–10)	17 (16–18)	23 (22–24)	22 (23–16)
Sex^a^ n (%)								
Female	6985 (45.7)	3751 (56.5)	36,937 (85.4)	47,673 (73.1)	31,630 (45.7)	17,901 (57.6)	183,113 (86.1)	232,644 (74.3)
Male	8308 (54.3)	2888 (43.5)	6304 (14.6)	17,500 (26.9)	37,559 (54.3)	13,170 (42.4)	29,562 (13.9)	80,291 (25.7)
Area of birth n (%)								
Sweden	14,616 (95.6)	6088 (91.7)	39,775 (92.0)	60,479 (92.8)	65,693 (94.9)	28,105 (90.5)	183,043 (86.1)	276,841 (88.5)
Europe	306 (2.0)	280 (4.2)	2134 (4.9)	2720 (4.2)	1465 (2.1)	1331 (4.3)	14,737 (6.9)	17,533 (5.6)
Other^b^	371 (2.4)	271 (4.1)	1332 (3.1)	1974 (3.0)	2031 (2.9)	1635 (5.3)	14 895 (7.0)	18,561 (5.9)
Decade at index diagnosis n (%)								
1958–1970	2436 (15.9)	928 (14.0)	3064 (7.1)	6428 (9.9)	5332 (7.7)	2641 (8.5)	121 56 (5.7)	20,129 (6.4)
1971–1980	2134 (14.0)	1105 (16.6)	6260 (14.5)	9499 (14.6)	10,504 (15.2)	5505 (17.7)	31,179 (14.7)	47,188 (15.1)
1981–1990	2223 (14.5)	1054 (15.9)	6634 (15.3)	9911 (15.2)	11,031 (15.9)	5250 (16.9)	33,109 (15.6)	49,390 (15.8)
1991–2000	2654 (17.4)	1048 (15.8)	5468 (12.6)	9170 (14.1)	13,233 (19.1)	5230 (16.8)	27,285 (12.8)	45,748 (14.6)
2001–2010	2630 (17.2)	1146 (17.3)	6482 (15.0)	10,258 (15.7)	13,102 (18.9)	5705 (18.4)	32,341 (15.2)	51,148 (16.3)
2011–2021	3216 (21.0)	1358 (20.5)	15,333 (35.5)	19,907 (30.5)	15,987 (23.1)	6740 (21.7)	76,605 (36.0)	99,332 (31.7)
Living conditions at index								
Na for living area/condition n (%)	568 (3.7)	136 (2.0)	240 (0.6)	944 (1.4)	–	–	–	–
Part of Sweden^c^ n (%)								
North	1929 (12.6)	835 (12.6)	5226 (12.1)	7990 (12.3)	8708 (12.6)	3880 (12.5)	25,814 (12.1)	38,402 (12.3)
Middle	5378 (35.2)	2216 (33.4)	13,920 (32.2)	21,514 (33.0)	26,167 (37.8)	10,825 (34.8)	69,091 (32.5)	106,083 (33.9)
South	7418 (48.5)	3452 (52.0)	23,855 (55.2)	34,725 (53.3)	34,314 (49.6)	16,366 (52.7)	117,770 (55.4)	168,450 (53.8)
Inhabitant/km^2^ in municipal n (%)								
<16	1842 (12.0)	797 (12.0)	3940 (9.1)	6579 (10.1)	8342 (12.1)	3715 (12.0)	19,423 (9.1)	31,480 (10.1)
16–2241	11,132 (72.8)	4972 (74.9)	33,156 (76.7)	49,260 (75.6)	52,947 (76.5)	23,971 (77.1)	164,280 (77.2)	241,198 (77.1)
≥2241	1751 (11.4)	734 (11.1)	5905 (13.7)	8390 (12.9)	7900 (11.4)	3385 (10.9)	28,972 (13.6)	40,257 (12.9)
Median income^d^ in municipal (SEK) n (%)								
>363,000	1284 (8.4)	497 (7.5)	2757 (6.4)	4538 (7.0)	6345 (9.2)	2455 (7.9)	13,690 (6.4)	22,490 (7.2)
286,000–363,000	11,773 (77.0)	5272 (79.4)	36,305 (84.0)	53,350 (81.9)	55,102 (79.6)	25,156 (81.0)	179,540 (84.4)	259,798 (83.0)
<286,000	1668 (10.9)	734 (11.1)	3939 (9.1)	6341 (9.7)	7742 (11.2)	3460 (11.1)	19,445 (9.1)	30,647 (9.8)
Gini coefficient^e^ n (%)								
>0.42	1581 (10.3)	588 (8.9)	4646 (10.7)	6815 (10.5)	7250 (10.5)	2730 (8.8)	22,831 (10.7)	32,811 (10.5)
0.31–0.42	11,401 (74.6)	5144 (77.5)	34,571 (79.9)	51,116 (78.4)	54,106 (78.2)	24,726 (79.6)	171,236 (80.5)	250,068 (79.9)
<0.31	1743 (11.4)	771 (11.6)	3784 (8.8)	6298 (9.7)	7833 (11.3)	3615 (11.6)	18,608 (8.7)	30,056 (9.6)
Proximity to hospital n (%)								
<30 km (19 miles)	11,511 (75.3)	5056 (76.2)	35,684 (82.5)	52,251 (80.2)	54,240 (78.4)	24,246 (78.0)	176,538 (83.0)	255,024 (81.5)
30–100 km	3066 (20.0)	1380 (20.8)	7091 (16.4)	11,537 (17.7)	14,294 (20.7)	6530 (21.0)	35,048 (16.5)	55,872 (17.9)
>100 km (62 miles)	148 (1.0)	67 (1.0)	226 (0.5)	441 (0.7)	655 (0.9)	295 (0.9)	1089 (0.5)	2039 (0.7)
Index cancer diagnosis n (%)								
Leukaemia	4693 (30.7)	645 (9.7)	772 (1.8)	6110 (9.4)	–	–	–	–
Acute lymphoblastic leukaemia	3072 (20.1)	307 (4.6)	240 (0.6)	3619 (5.6)	–	–	–	–
Acute myeloid leukaemia	688 (4.5)	226 (3.4)	396 (0.9)	1310 (2.0)	–	–	–	–
Hodgkin lymphoma	405 (2.6)	567 (8.5)	1159 (2.7)	2131 (3.3)	–	–	–	–
Non-Hodgkin lymphoma	674 (4.4)	279 (4.2)	430 (1.0)	1383 (2.1)	–	–	–	–
CNS Tumours	3981 (26.0)	849 (12.8)	1371 (3.2)	6201 (9.5)	–	–	–	–
Soft tissue sarcoma	594 (3.9)	219 (3.3)	311 (0.7)	1124 (1.7)	–	–	–	–
Bone tumours	685 (4.5)	468 (7.0)	406 (0.9)	1559 (2.4)	–	–	–	–
Gastrointestinal	433 (2.8)	348 (5.2)	830 (1.9)	1611 (2.5)	–	–	–	–
Kidney and urorenal tract	919 (6.0)	59 (0.9)	176 (0.4)	1154 (1.8)	–	–	–	–
Male reproductive organs	179 (1.2)	303 (4.6)	1689 (3.9)	2171 (3.3)	–	–	–	–
Prostate	1 (0.01)	4 (0.1)	6 (0.01)	11 (0.02)	–	–	–	–
Testis	162 (1.1)	286 (4.3)	1582 (3.7)	2030 (3.1)	–	–	–	–
Female reproduction organ	193 (1.3)	1491 (22.5)	31,728 (73.4)	33,412 (51.3)	–	–	–	–
Cervix	8 (0.1)	940^f^ (14.2)	29,573^f^ (68.4)	30,521 (46.8)	–	–	–	–
Breast	6 (0.04)	17 (0.3)	172 (0.4)	195 (0.3)	–	–	–	–
Eye	431 (2.8)	17 (0.3)	32 (0.1)	480 (0.7)	–	–	–	–
Thyroid and other endocrine glands	677 (4.4)	614 (9.2)	1482 (3.4)	2773 (4.3)	–	–	–	–
Skin	192 (1.3)	443 (6.7)	1946 (4.5)	2581 (4.0)	–	–	–	–
Other malignancies	1206 (7.9)	290 (4.4)	635 (1.5)	2131 (3.3)	–	–	–	–
Cancer treatment^g^ n (%)	8678 (56.7)	2351 (35.4)	4920 (11.4)	15,949 (24.5)				
Radiotherapy	5199 (59.9)	1363 (58.0)	2796 (56.8)	9358 (58.7)	–	–	–	–
Anthracycline	4227 (48.7)	1084 (46.1)	1693 (34.4)	7004 (43.9)	–	–	–	–
Other drugs	7580 (87.3)	1956 (83.2)	4010 (81.5)	13,546 (84.9)	–	–	–	–

Patients included were between <1 years and 24 years at the time of the index cancer diagnosis and their 1:5 matched controls regarding age, sex and place of residence. The factors that are considered high and low are based on the 10th and 90th percentiles.

Abbreviations: CAYAs, children, adolescents, and young adults; IQR, interquartile range; SD, standard deviation; CNS, central nervous system; –, not applicable; n, numbers; NA, not available.

All the cancer in Sweden between January 1958 and December 2021 in patients under the age of 25 years.

a Sex assigned at birth.

b Other = North America 0.1%, Soth America 0.4%, Africa 1.0%, Asia 3.7%, Russia 0.02%, Oceanian 0.03%, and unknown 0.03%.

c North (Norrland) 11.4%, Middle (Svealand) 40.8%, and South (Gotaland) 47.8% of Sweden's population.

d Median income in municipal is high and low according to 10th and 90th percentiles.

e Gini coefficient ranges from 0 to 1, indicates income inequality within each municipality, with a higher value signifying greater unevenness and are calculated high and low according to 10th and 90th percentiles.

f Including cervical intraepithelial neoplasia (CIN), and high-grade squamous intraepithelial lesion (HSIL).

g Treatment for leukaemia, CNS malignancies, Hodgkin's lymphoma, non-Hodgkins's lymphoma, and testis cancer according to standard protocols.

**Table 2:** Occurrence of morbidities, mortality, and socioeconomic factors of 65,173 in child, adolescent, and young adult patients with cancer and their 312,935 matched controls.

Age group at index cancer	CAYAs n = 65,173				Controls n = 312,935				
	<1–14	15–18	19–24	Total	<1–14	15–18	19–24	Total	p-value
n (%)	15,293 (23.5)	6639 (10.2)	43,241 (66.3)	65,173	69,189 (22.1)	31,071 (9.9)	212,675 (68.0)	312,935	
Age at index date, years median (IQR)	5 (2–10)	17 (16–18)	23 (21–24)	21 (15–23)	5 (2–10)	17 (16–18)	23 (22–24)	22 (23–16)	<0.0001
Follow up									
Years of follow up median (IQR)	10.0 (1.6–26.7)	16.1 (3.7–34.3)	16.0 (5.1–35.1)	14.6 (4.2–33.4)	24.9 (11.8–39.1)	26.9 (12.2–41.0)	19.5 (6.9–37.2)	22.0 (8.1–38.0)	<0.0001
Age at study end years n (%)									
0–5	2284 (14.9)	–	–	2284 (3.5)	1971 (2.8)	–	–	1971 (0.6)	<0.0001
6–10	2268 (14.8)	–	–	2268 (3.5)	4408 (6.4)	–	–	4408 (1.4)	<0.0001
11–20	3958 (25.9)	1583 (23.8)	225 (0.5)	5766 (8.8)	13,398 (19.4)	2103 (6.8)	256 (0.1)	15,757 (5.0)	<0.0001
21–30	2373 (15.5)	1425 (21.5)	13,199 (30.5)	16,997 (26.1)	13,536 (19.6)	6369 (20.5)	54,938 (25.8)	74,843 (23.9)	<0.0001
31–40	1887 (12.3)	931 (14.0)	9052 (20.9)	11,870 (18.2)	12,742 (18.4)	5265 (16.9)	45,500 (21.4)	63,507 (20.3)	<0.0001
41–50	1330 (8.7)	916 (13.8)	5482 (12.7)	7728 (11.9)	10,816 (15.6)	5494 (17.7)	28,354 (13.3)	44,664 (14.3)	<0.0001
51–60	759 (5.0)	808 (12.2)	6173 (14.3)	7740 (11.9)	8792 (12.7)	5255 (16.9)	32,564 (15.3)	46,611 (14.9)	<0.0001
61–70	349 (2.3)	710 (10.7)	5897 (13.6)	6956 (10.7)	3031 (4.4)	4945 (15.9)	32,119 (15.1)	40,095 (12.8)	<0.0001
≥71	85 (0.6)	265 (4.0)	3213 (7.4)	3563 (5.5)	495 (0.7)	1640 (5.3)	18,944 (8.9)	21,079 (6.7)	<0.0001
Median age at study end (IQR)	16.7 (8.2–32.6)	32.9 (20.4–51.2)	38.7 (28.2–57.2)	33.4 (24.7–52.6)	30.8 (18.2–45.5)	43.5 (29.0–58.0)	42.0 (29.7–59.3)	39.2 (28.0–56.3)	<0.0001
Highest level of Education n (%)									
Elementary school 9 years	2389 (15.6)	1760 (26.5)	5974 (13.8)	10,123 (15.5)	8340 (12.1)	4265 (13.7)	20,037 (9.4)	32,642 (10.4)	<0.0001
Upper secondary school	3224 (21.1)	2474 (37.3)	19,315 (44.7)	25,013 (38.4)	24,119 (34.9)	13,922 (44.8)	88,581 (41.7)	126,622 (40.5)	<0.0001
University	2719 (17.8)	1810 (27.3)	16,734 (38.7)	21,263 (32.6)	21,099 (30.5)	11,945 (38.4)	96,373 (45.3)	129,417 (41.4)	<0.0001
Postgraduate education	41 (0.3)	39 (0.6)	232 (0.5)	312 (0.5)	432 (0.6)	247 (0.8)	1590 (0.7)	2269 (0.7)	<0.0001
Na	6920 (45.2)	556 (8.4)	986 (2.3)	8462 (13.0)	15,199 (22.0)	692 (2.2)	6094 (2.9)	21,985 (7.0)	<0.0001
Civil status^a^ n (%)									
Unmarried	12 583 (82.3)	4202 (63.3)	22,210 (51.4)	38,995 (59.8)	48,827 (70.6)	16,341 (52.6)	101,211 (47.6)	166,379 (53.2)	<0.0001
Married or registered partner	1550 (10.1)	1430 (21.5)	12,315 (28.5)	15,295 (23.5)	14,641 (21.2)	9667 (31.1)	72,303 (34.0)	96,611 (30.9)	<0.0001
Na	1160 (7.6)	1007 (15.2)	8716 (20.2)	10 883 (16.7)	5721 (8.3)	5063 (16.3)	39 159 (18.4)	49,945 (16.0)	<0.0001
Subsequent malignancy^b^ n (%)									
All malignancy	1573 (10.3)	482 (7.3)	1618 (3.7)	3673 (5.6)	574 (0.8)	623 (2.0)	4913 (2.3)	6110 (2.0)	<0.0001
Lip oral cavity pharynx	40 (0.3)	25 (0.4)	53 (0.1)	118 (0.2)	13 (0.0)	20 (0.1)	130 (0.1)	163 (0.1)	<0.0001
Oesophagus stomach small intestine	21 (0.1)	6 (0.1)	16 (0.0)	43 (0.1)	4 (0.0)	6 (0.0)	30 (0.0)	40 (0.0)	<0.0001
Colon	13 (0.1)	4 (0.1)	23 (0.1)	40 (0.1)	4 (0.0)	5 (0.0)	69 (0.0)	78 (0.0)	<0.0001
Liver gallbladder	7 (0.0)	2 (0.0)	24 (0.1)	33 (0.1)	6 (0.0)	8 (0.0)	46 (0.0)	60 (0.0)	<0.0001
Pancreas	3 (0.0)	4 (0.1)	21 (0.0)	28 (0.0)	10 (0.0)	7 (0.0)	61 (0.0)	78 (0.0)	0.018
Other gastrointestinal	12 (0.1)	1 (0.0)	8 (0.0)	21 (0.0)	1 (0.0)	0 (0.0)	3 (0.0)	4 (0.0)	–
Mouth airways lungs	34 (0.2)	16 (0.2)	52 (0.1)	102 (0.2)	11 (0.0)	9 (0.0)	98 (0.0)	118 (0.0)	<0.0001
Bone connective soft tissue	91 (0.6)	21 (0.3)	31 (0.1)	143 (0.2)	3 (0.0)	2 (0.0)	14 (0.0)	19 (0.0)	<0.0001
Skin cancers	219 (1.4)	142 (2.1)	790 (1.8)	1151 (1.8)	439 (0.6)	499 (1.6)	3841 (1.8)	4779 (1.5)	<0.0001
Breast	9 (0.1)	6 (0.1)	35 (0.1)	50 (0.1)	13 (0.0)	11 (0.0)	131 (0.1)	155 (0.0)	0.0088
Female reproductive	18 (0.1)	11 (0.2)	53 (0.1)	82 (0.1)	10 (0.0)	9 (0.0)	111 (0.1)	130 (0.0)	<0.0001
Cervix	1 (0.0)	2 (0.0)	5 (0.0)	8 (0.0)	0 (0.0)	1 (0.0)	14 (0.0)	15 (0.0)	–
Prostate	10 (0.1)	1 (0.0)	8 (0.0)	19 (0.0)	6 (0.0)	10 (0.0)	21 (0.0)	37 (0.0)	0.0017
Testis	9 (0.1)	5 (0.1)	9 (0.0)	23 (0.0)	3 (0.0)	2 (0.0)	1 (0.0)	6 (0.0)	–
Other genitourinary	26 (0.2)	6 (0.1)	16 (0.0)	48 (0.1)	13 (0.0)	7 (0.0)	73 (0.0)	93 (0.0)	<0.0001
Kidney and bladder	289 (1.9)	49 (0.7)	73 (0.2)	411 (0.6)	9 (0.0)	7 (0.0)	50 (0.0)	66 (0.0)	<0.0001
CNS	79 (0.5)	16 (0.2)	20 (0.0)	115 (0.2)	5 (0.0)	4 (0.0)	30 (0.0)	39 (0.0)	<0.0001
Thyroid, and other endocrine glands	194 (1.3)	75 (1.1)	117 (0.3)	386 (0.6)	8 (0.0)	6 (0.0)	67 (0.0)	81 (0.0)	<0.0001
Lymphoma	270 (1.8)	34 (0.5)	59 (0.1)	363 (0.6)	4 (0.0)	3 (0.0)	42 (0.0)	49 (0.0)	<0.0001
Leukaemia	106 (0.7)	42 (0.6)	95 (0.2)	243 (0.4)	21 (0.0)	10 (0.0)	194 (0.1)	225 (0.1)	<0.0001
Benign meningioma	266 (1.7)	46 (0.7)	106 (0.2)	418 (0.6)	17 (0.0)	14 (0.0)	119 (0.1)	150 (0.0)	<0.0001
All other specified neoplasms	1573 (10.3)	482 (7.3)	1618 (3.7)	3673 (5.6)	574 (0.8)	623 (2.0)	4913 (2.3)	6110 (2.0)	<0.0001
Relapse of index cancer n (%)									
>5 years	4141 (27.1)	1117 (16.8)	2502 (5.8)	7760 (11.9)	–	–	–	–	–
Cardiovascular diseases n (%)									
All cardiovascular diseases	1963 (12.8)	1345 (20.3)	7695 (17.8)	11,003 (16.9)	6351 (9.2)	4937 (15.9)	33,844 (15.9)	45,132 (14.4)	<0.0001
Coronary artery diseases	89 (0.6)	157 (2.4)	967 (2.2)	1213 (1.9)	510 (0.7)	648 (2.1)	4118 (1.9)	5276 (1.7)	0.0018
Pulmonary embolism	75 (0.5)	72 (1.1)	408 (0.9)	555 (0.9)	199 (0.3)	201 (0.6)	1284 (0.6)	1684 (0.5)	<0.0001
Myo- endo- and pericardial	44 (0.3)	19 (0.3)	74 (0.2)	137 (0.2)	179 (0.3)	101 (0.3)	359 (0.2)	639 (0.2)	0.79
Arrhythmias	285 (1.9)	231 (3.5)	1600 (3.7)	2116 (3.2)	1399 (2.0)	1139 (3.7)	7402 (3.5)	9940 (3.2)	0.36
Heart failure and cardiomyopathy	195 (1.3)	133 (2.0)	543 (1.3)	871 (1.3)	255 (0.4)	329 (1.1)	1987 (0.9)	2571 (0.8)	<0.0001
Valvular diseases	109 (0.7)	90 (1.4)	330 (0.8)	529 (0.8)	232 (0.3)	182 (0.6)	1353 (0.6)	1767 (0.6)	<0.0001
Cerebrovascular	384 (2.5)	213 (3.2)	1001 (2.3)	1598 (2.5)	438 (0.6)	451 (1.5)	3511 (1.7)	4400 (1.4)	<0.0001
Hypertension	398 (2.6)	383 (5.8)	2748 (6.4)	3529 (5.4)	1642 (2.4)	1853 (6.0)	13,507 (6.4)	17,002 (5.4)	0.86
Pulmonary n (%)									
All Pulmonary Diseases	5742 (37.5)	1771 (26.7)	8775 (20.3)	16,288 (25.0)	17,404 (25.2)	6139 (19.8)	35,535 (16.7)	59,078 (18.9)	<0.0001
Chronic lower respiratory disease	49 (0.3)	75 (1.1)	761 (1.8)	885 (1.4)	150 (0.2)	221 (0.7)	1859 (0.9)	2230 (0.7)	<0.0001
Interstitial lung disease	62 (0.4)	19 (0.3)	92 (0.2)	173 (0.3)	34 (0.0)	50 (0.2)	259 (0.1)	343 (0.1)	<0.0001
Other health related cause n (%)									
Infectious and parasitic	6498 (42.5)	1910 (28.8)	8731 (20.2)	17,139 (26.3)	12,480 (18.0)	5240 (16.9)	32,383 (15.2)	50,103 (16.0)	<0.0001
Blood and blood-forming organs	3189 (20.9)	858 (12.9)	2688 (6.2)	6735 (10.3)	1757 (2.5)	1109 (3.6)	8101 (3.8)	10,967 (3.5)	<0.0001
Thyroid and other endocrine glands	2697 (17.6)	1231 (18.5)	5844 (13.5)	9772 (15.0)	5375 (7.8)	3020 (9.7)	23,876 (11.2)	32,271 (10.3)	<0.0001
Mental illness	2348 (15.4)	1332 (20.1)	8464 (19.6)	12,144 (18.6)	11,915 (17.2)	5720 (18.4)	35,175 (16.5)	52,810 (16.9)	<0.0001
Neurological	2266 (14.8)	950 (14.3)	4844 (11.2)	8060 (12.4)	5413 (7.8)	2953 (9.5)	19,385 (9.1)	27,751 (8.9)	<0.0001
Eye	3853 (25.2)	1447 (21.8)	8790 (20.3)	14,090 (21.6)	11,017 (15.9)	5962 (19.2)	42,562 (20.0)	59,541 (19.0)	<0.0001
Ear	3003 (19.6)	710 (10.7)	4141 (9.6)	7854 (12.1)	7305 (10.6)	3035 (9.8)	20,059 (9.4)	30,399 (9.7)	<0.0001
Gastrointestinal	4420 (28.9)	2133 (32.1)	12,338 (28.5)	18,891 (29.0)	16,226 (23.5)	8280 (26.6)	53,388 (25.1)	77,894 (24.9)	<0.0001
Skin	4231 (27.7)	1805 (27.2)	10,653 (24.6)	16,689 (25.6)	14,773 (21.4)	7368 (23.7)	47,375 (22.3)	69,516 (22.2)	<0.0001
Musculoskeletal	3881 (25.4)	2136 (32.2)	14,319 (33.1)	20,336 (31.2)	19,241 (27.8)	10,515 (33.8)	66,996 (31.5)	96,752 (30.9)	0.15
kidney and genitourinary	3756 (24.6)	2617 (39.4)	24,716 (57.2)	31,089 (47.7)	18,147 (26.2)	11,030 (35.5)	87,915 (41.3)	117,092 (37.4)	<0.0001
External affliction^c^	290 (1.9)	189 (2.8)	1420 (3.3)	1899 (2.9)	476 (0.7)	673 (2.2)	5525 (2.6)	6674 (2.1)	<0.0001
Remaining intact teeth mean (SD)	22.42 (7.70)	19.75 (9.04)	20.18 (9.04)	20.40 (8.92)	22.31 (7.53)	20.00 (8.69)	20.11 (8.93)	20.46 (8.74)	0.18
Hospital care n (%)	13,024 (85.2)	5830 (87.8)	37,724 (87.2)	56,578 (86.8)	40,324 (58.3)	22,574 (72.7)	169,211 (79.6)	232,109 (74.2)	<0.0001
Total Hospital care days median (IQR)	67.0 (25.0–124.0)	28.0 (11.0–80.0)	12.0 (5.0–31.0)	19.0 (7.0–59.0)	5.0 (2.0–12.0)	8.0 (3.0–17.0)	9.0 (4.0–18.0)	8.0 (4.0–17.0)	.
>5 years after index days median (IQR)	10.0 (3.0–34.0)	10.0 (4.0–28.0)	7.0 (3.0–18.0)	8.0 (3.0–21.0)	6.0 (3.0–13.0)	8.0 (4.0–16.0)	7.0 (3.0–15.0)	7.0 (3.0–14.0)	.
Total ICU hours median (IQR)	41.0 (19.0–123.7)	43.5 (18.7–109.5)	29.2 (15.0–86.9)	36.2 (17.5–104.6)	24.7 (12.6–64.4)	26.7 (14.7–68.0)	26.0 (14.6–71.0)	25.8 (14.2–69.5)	.
>5 years after index hours median (IQR)	34.6 (17.9–116.4)	40.3 (17.4–100.5)	33.3 (17.7–97.2)	34.7 (17.7–100.4)	24.7 (12.7–62.1)	31.1 (16.3–77.4)	32.8 (17.3–90.5)	30.0 (16.1–82.3)	0.0061
Medical leave days n (%) Mean (SD)	3750 (24.5)	3314 (49.9)	27 353 (63.3)	34,417 (52.8)	23,898 (34.5)	16,125 (51.9)	121,215 (57.0)	161,238 (51.5)	<0.0001
Sick leave >180 days. n (%)	802 (5.2)	897 (13.5)	8090 (18.7)	9789 (15.0)	4644 (6.7)	3835 (12.3)	29,343 (13.8)	37,822 (12.1)	<0.0001
Total sick leave + disability pension days median (IQR)	226.4 (44.5–1645.0)	245.0 (57.0–1138.2)	165.0 (43.0–731.2)	177.0 (44.0–843.5)	93.5 (26.5–375.0)	115.0 (31.0–524.2)	120.5 (32.0–585.8)	115.5 (31.0–537.9)	<0.0001
Years in study n (%)									
0–5	6061 (39.6)	1889 (28.5)	10 642 (24.6)	18,592 (28.5)	7507 (10.8)	3063 (9.9)	41,930 (19.7)	52,500 (16.8)	<0.0001
6–10	1597 (10.4)	692 (10.4)	6248 (14.4)	8537 (13.1)	7200 (10.4)	3159 (10.2)	30,358 (14.3)	40,717 (13.0)	0.55
11–20	2347 (15.3)	1087 (16.4)	6962 (16.1)	10 396 (16.0)	13 233 (19.1)	5904 (19.0)	35,422 (16.7)	54,559 (17.4)	<0.0001
21–30	2161 (14.1)	865 (13.0)	5175 (12.0)	8201 (12.6)	13,556 (19.6)	5150 (16.6)	26,900 (12.6)	45,606 (14.6)	<0.0001
31–40	1544 (10.1)	882 (13.3)	6306 (14.6)	8732 (13.4)	11,296 (16.3)	5492 (17.7)	33 374 (15.7)	50,162 (16.0)	<0.0001
41–50	924 (6.0)	805 (12.1)	5742 (13.3)	7471 (11.5)	10,239 (14.8)	5431 (17.5)	31,581 (14.8)	47,251 (15.1)	<0.0001
51–60	499 (3.3)	335 (5.0)	2033 (4.7)	2867 (4.4)	5333 (7.7)	2442 (7.9)	12,357 (5.8)	20,132 (6.4)	<0.0001
>61	160 (1.0)	83 (1.3)	133 (0.3)	376 (0.6)	825 (1.2)	430 (1.4)	753 (0.4)	2008 (0.6)	0.061
Mortality									
All-cause mortality n (%)	5531 (36.2)	1763 (26.6)	4390 (10.4)	11,684 (17.9)	1063 (1.5)	985 (3.2)	5970 (2.8)	8018 (2.6)	<0.0001
Proportion censored n (%)	9762 (63.8)	4876 (73.4)	37,851 (89.6)	53,489 (82.1)	68,126 (98.5)	30,086 (96.8)	206,705 (97.2)	304,917 (97.4)	<0.0001
Female n (%)	2469 (44.6)	687 (39.0)	2438 (55.5)	5594 (47.9)	310 (29.2)	423 (44.1)	4460 (74.7)	5193 (64.8)	<0.0001
Age median (IQR)	7.6 (3.6–12.9)	18.4 (17.0–21.5)	25.8 (22.8–8.5)	18.2 (8.1–25.3)	36.2 (23.8–50.7)	51.7 (36.5–63.6)	57.9 (43.4–67.9)	54.7 (38.6–66.0)	<0.0001
Year after index median (IQR)	1.0 (0.4–2.7)	1.5 (0.5–4.8)	3.5 (0.9–26.6)	1.6 (0.5–6.2)	29.4 (17.4–42.9)	35.0 (19.9–47.1)	35.8 (21.3–45.7)	35.0 (20.1–45.5)	<0.0001
>5 years after index median age (IQR)	20.1 (13.3–31.1)	37.6 (25.1–56.1)	52.1 (35.7–65.0)	42.2 (27.3–60.3)	37.9 (25.5–51.7)	53.3 (40.2–64.5)	59.4 (46.8–68.5)	56.4 (42.4–66.7)	<0.0001
Cancer specific mortality n (%)	3211 (21.0)	1029 (15.5)	2157 (5.0)	6397 (9.8)	19 (0.0)	27 (0.1)	229 (0.1)	275 (0.1)	<0.0001
Age median (IQR)	6.9 (3.4–11.8)	18.2 (16.9–19.7)	23.7 (21.7–25.9)	16.3 (6.8–22.4)	57.2 (52.6–65.1)	64.9 (56.9–70.1)	66.6 (60.1–72.4)	65.7 (58.6–71.8)	<0.0001
Year after index median (IQR)	0.8 (0.3–2.1)	1.1 (0.4–2.8)	1.4 (0.6–3.5)	1.1 (0.4–2.5)	47.5 (43.4–56.0)	47.1 (40.5–53.1)	44.4 (37.7–50.3)	44.5 (38.0–50.8)	<0.0001
>5 years after index median age (IQR)	17.0 (11.2–22.3)	24.8 (22.8–29.4)	31.9 (29.0–42.4)	28.2 (21.1–33.7)	57.2 (52.6–65.1)	64.9 (56.9–70.1)	66.6 (60.3–72.5)	65.8 (58.9–71.8)	<0.0001
Cardiovascular mortality n (%)	433 (2.8)	199 (3.0)	620 (1.4)	1252 (1.9)	216 (0.3)	303 (1.0)	1810 (0.9)	2329 (0.7)	<0.0001
Age median (IQR)	9.5 (4.0–14.7)	27.8 (17.8–56.0)	52.9 (29.3–64.9)	28.9 (14.2–58.1)	48.4 (34.2–58.0)	58.2 (47.8–66.6)	62.1 (52.0–69.6)	60.3 (49.6–68.5)	<0.0001
Year after index median (IQR)	1.5 (0.4–5.7)	11.5 (1.1–39.6)	31.6 (7.3–43.0)	10.3 (1.1–37.6)	39.9 (27.2–49.1)	41.2 (31.1–50.3)	39.8 (29.8–47.4)	40.0 (29.9–47.9)	<0.0001
>5 years after index median age (IQR)	29.0 (15.5–48.9)	55.0 (42.8–63.4)	59.0 (48.5–67.2)	55.6 (42.1–65.1)	49.3 (36.3–58.6)	58.3 (48.1–66.6)	62.3 (52.8–69.8)	60.6 (50.5–68.7)	<0.0001
External affliction^c^ n (%)	99 (0.6)	74 (1.1)	366 (0.8)	539 (0.8)	492 (0.7)	315 (1.0)	1394 (0.7)	2201 (0.7)	<0.0001
Age median (IQR)	23.9 (17.0–31.0)	29.5 (21.2–46.1)	40.0 (29.5–51.3)	36.0 (24.9–48.4)	27.6 (20.6–38.9)	35.1 (24.7–47.2)	38.7 (29.0–50.7)	35.7 (26.7–48.0)	0.30
Year after index median (IQR)	16.7 (6.7–25.7)	13.3 (4.6–29.3)	17.1 (7.3–29.1)	16.8 (6.8–28.1)	20.7 (13.7–32.3)	18.2 (8.1–30.5)	16.7 (6.8–28.8)	18.3 (8.2–29.8)	0.022
>5 years after index median age (IQR)	27.4 (21.8–33.5)	37.7 (28.2–50.5)	44.6 (36.1–53.4)	40.6 (30.8–51.5)	28.4 (21.9–40.0)	40.0 (29.8–49.9)	43.1 (34.0–53.7)	39.5 (30.5–50.4)	0.19

p-values apply between all CAYAs and all controls, regardless of age group. Abbreviations: CAYA, child, adolescent, and young adult; IQR, interquartile range; SD, standard deviation; CNS, central nervous system or Na, not applicable; n, numbers.

All the cancer patients in Sweden under the age of 25 years between January 1958 and December 2021.

a A Swedish citizen is registered as unmarried at birth or immigration, and this is changed to married and thereafter divorced or widowed in later life.

b Subsequent malignancy: >5 years after index date, for controls primary cancer after index date.

c Injuries, poisonings, suicide, and other consequences of external causes.

In 90 cases, only the year of mortality was documented. If the year matched the index date, December 30th was added; otherwise, June 30th was included. Additionally, for 2 cases where the time of death was registered a few weeks before the index date, it was adjusted to align with the same date.

The analysis was performed and validated independently by two researchers and presented as descriptive and comparative statistics using R 4.3.2, R Foundation (Vienna, Austria).

### Role of the funding source

The funders of the study played no role in study design, data collection, data analysis, data interpretation, or writing of the report.

## Results

### Study population and baseline characteristics

The study spanned a 63-year observation period, involving a total of 378,108 individuals, (74% females). Among the participants, 65,173 were CAYAs aged between <1 and 24 years, who experienced an index cancer (1.24 million patient-years). A control group of 312,935 matched individuals was created (7.45 million person-years). Baseline demographic, index cancers, clinical, and socioeconomic characteristics are presented in Table 1.

**Fig. 1:**
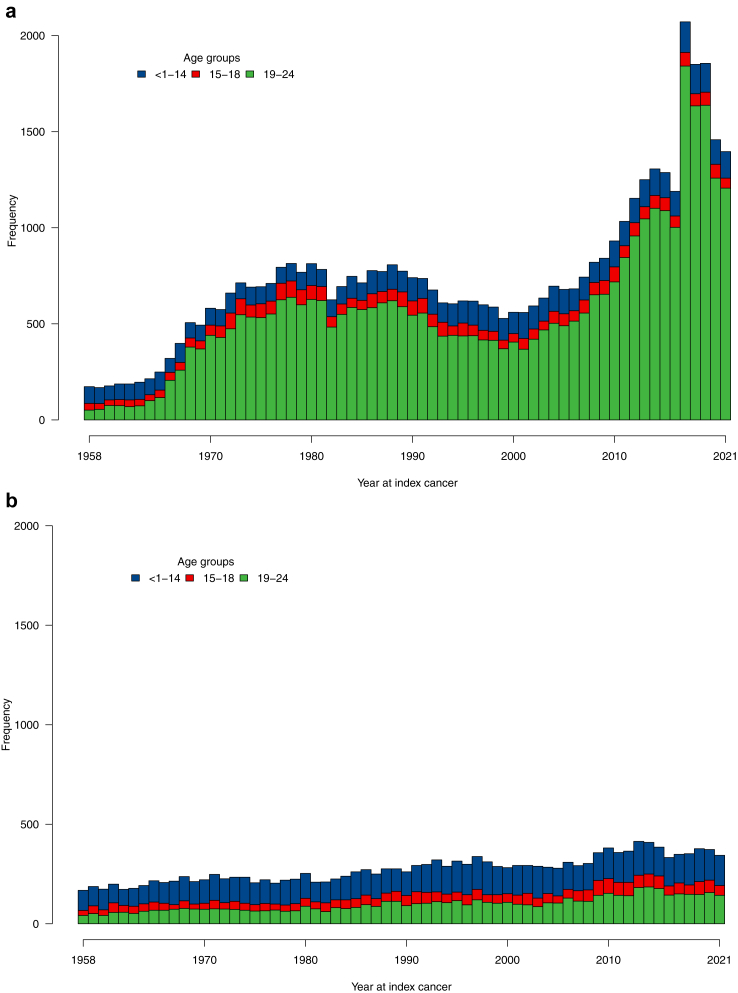
Distribution of 65,173 young cancer patients by age groups (<1–14, 15–18, and 19–24) over time in Sweden from 1958 to 2021. Panel (a) shows females, and panel (b) shows males. The higher number of young women is due to cervical cancer cases, including severe squamous cell abnormalities.

**Fig. 2:**
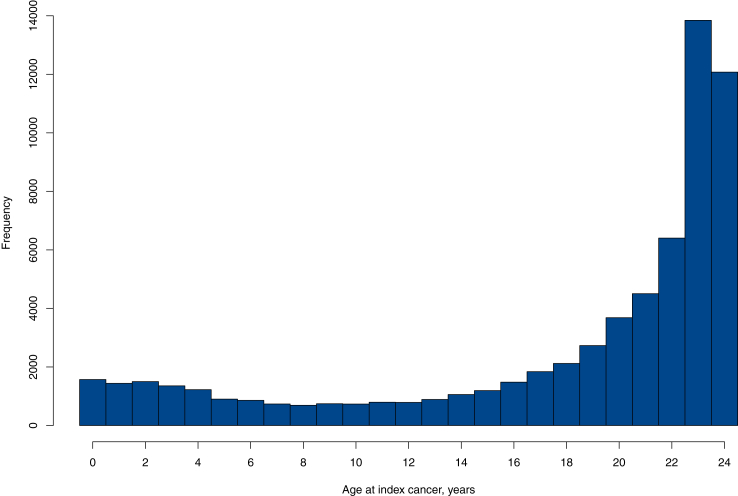
Age distribution of all 65,173 young individuals <25 years old with index cancer in Sweden 1958 and 2021. The majority were aged 19–24 (n = 43,241, 66.3%), followed by < 1–14 (n = 15,293, 23.5%) and the smallest group, 15–18 years old (n = 6639, 10.2%).

Index cancer frequencies was consistent across the decades, apart from 1958 to 1970 (9.9%) and 2011–2021 (30.5%) (Fig. 1). Most CAYAs (66.3%) were in the 19–24 age group, followed by the <1–14 age group (23.5%) (Fig. 2). Differences in sex distribution between the three age groups were significant.

The median follow-up was 14.6 (IQR 4.2–33.4) years for CAYAs, and 22.0 (8.1–38.0) for controls. At the end of the study, the median age of CAYAs was 33.4 (IQR 4.2–33.4) years and controls 39.2 (8.1–38.0), with an age span of <1–88 years in both groups (Table 2).

### Morbidity

**Fig. 3:**
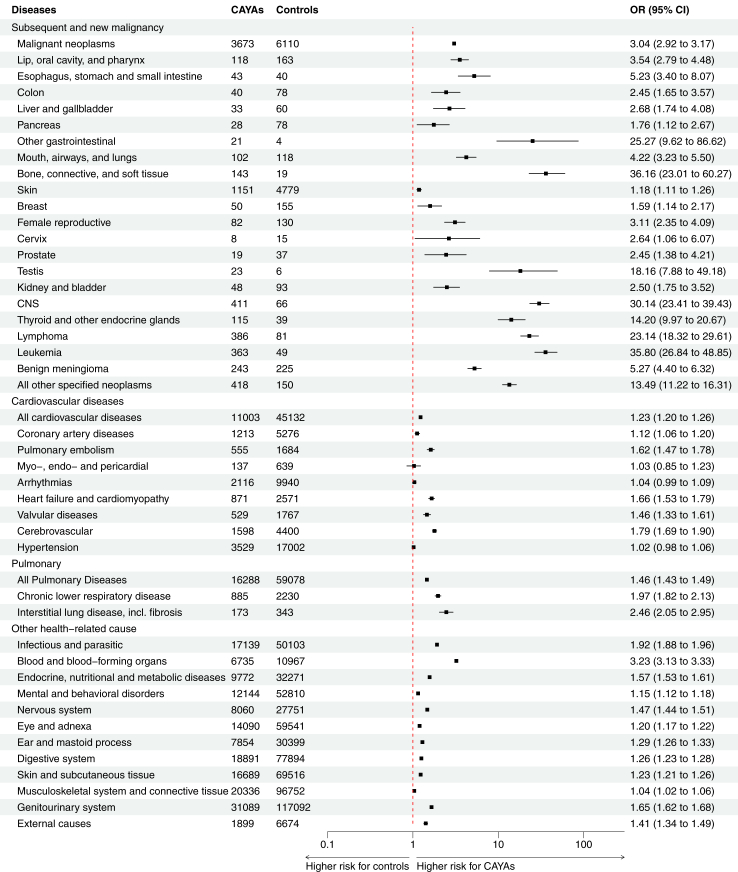
Odds ratio (OR) plot (95% confidence interval (CI) and logarithmic scale) comparing the risk for subsequent malignancy, cardiovascular, pulmonary, and other diseases between children, adolescents, and young adults with index cancer (CAYAs) and controls in Sweden from 1958 to 2021. The ORs were adjusted for age at index, sex, and part of Sweden at index. Abbreviations: CAYAs, children, adolescents, and young adults with index cancer. CNS, central nervous system.

Subsequent cancer was analysed in five-years survivors, and the risk for subsequent cancer was 3.04 times higher in CAYAs compared to controls (OR 95% CI 2.92–3.17, p < 0.0001). The highest subsequent cancer risk compared to controls was observed for bone, connective tissue, and soft tissue cancers, with a 36.16-times increased risk, followed by leukaemia and CNS cancers with 35.80 and 30.14-times higher risk, respectively. Among five-years survivors, 5.6% experienced a subsequent malignancy, and 11.9% had a relapse of their index cancer. Among controls with primary cancer (2.0%), the predominant type was skin cancer at 1.5%, followed by leukaemia at 0.1%, showing lower frequencies than in five-years survivors (Table 2 and Fig. 3).

CVD at any time after index was detected in 16.9% of the CAYAs, representing a 1.23-time elevated risk compared to controls (OR 95% CI 1.20–1.26, p < 0.0001). The most common CVDs were arrhythmia and hypertension, both with similar risks as controls, but many CVDs had a higher risk in CAYAs.

Pulmonary diseases were common (25%) among CAYAs and the risk was1.62-times higher compared to controls (OR 95% CI 1.47–1.78, p < 0.0001). It was common with kidney and genitourinary disease, seen among 47.7% of the CAYAs and with a 1.62-times higher risk (OR 95% CI 1.62–1.68, p < 0.0001), but there was no elevated risk for diabetes. In addition to the early cancer and the subsequent higher risk for various diseases thereafter, CAYAs had a 1.41- times higher risk for injury, poisoning, and other external events compared to controls (OR 95% CI 1.34–1.49, p < 0.0001). Frequencies and risk (OR) for all disease groups are listed in Table 2 and Fig. 3.

Throughout the study period, the CAYAs that were hospitalized had a higher median number of hospital days (19 vs 8) and hours in the intensive care unit, (ICU) (36 vs 26) compared to controls, and this difference persisted among five-years survivors (Table 2).

### Mortality

**Fig. 4:**
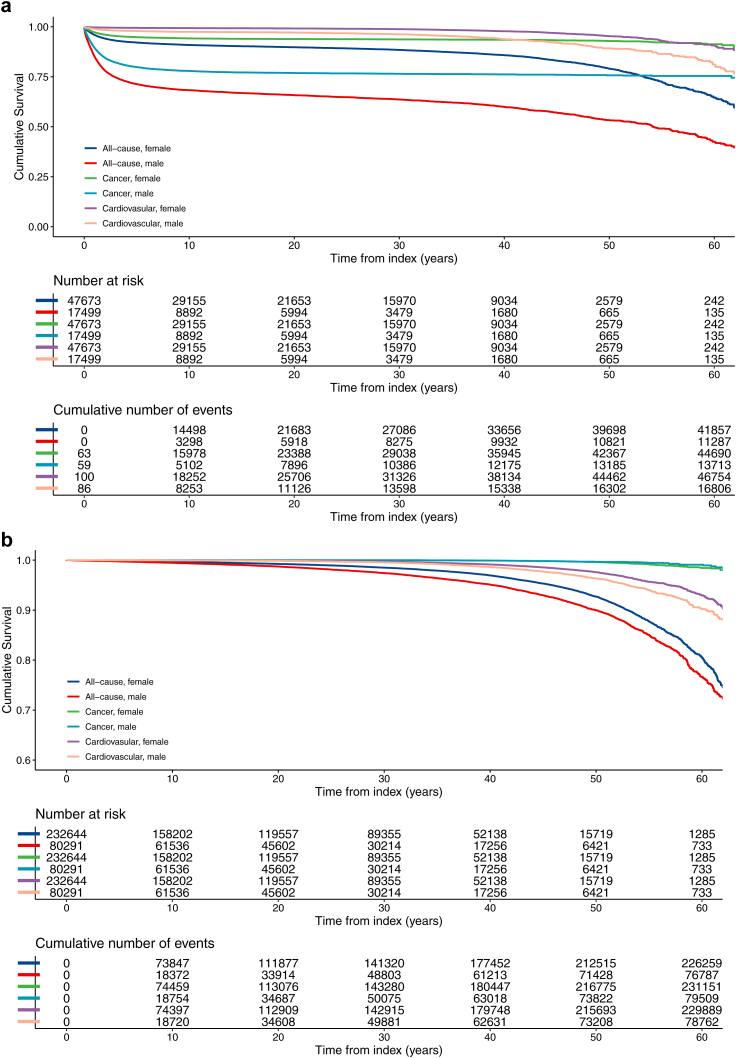
Cumulative all-cause, cancer-specific, and cardiovascular mortality of 65,173 females and male under <25 year of age with index cancer (CAYAs) (a), compared to their 312,935 matched controls (b) between 1958 and 2021.

CAYAs had a higher all-cause mortality; 17.9% compared to 2.6% in controls. The highest mortality was observed in children, 36.2% and decreasing with age groups (Table 2 and Appendix p 8). Females had a lower mortality in childhood and adolescent (44.6 vs 39.0%) but had slightly higher (55.5%) mortality than males in young adulthood (Table 2 and Fig. 4). The median time to death was 1.6 (IQR 0.5–6.2) years after index diagnosis, ranging from 1.0 (0.4–2.7) in children to 3.5 (0.9–26.6) years in young adults (Table 2 and Appendix p 8). The median age at death for CAYAs was 18.2 years (IQR 8.1–25.3), children being the youngest at 7.6 years (3.6–12.9) and increasing to 25.8 years (22.8–48.5) among young adults. Controls outlived CAYAs with 33.4 years. Among the five-years survivors, the median age at all-cause mortality was 42.2 (IQR 27.3–60.3) years, dying 14.2 years before the controls (Table 2).

#### Cancer specific and cardiovascular mortality

CAYAs had a higher cancer mortality (9.8% compared to 0.1% in controls) of their index–or subsequent cancer, and at a median age of 16.3 (IQR 6.8–22.4) years, 1.1 (0.4–2.5) years after index. Five-years survivors died of cancer at the age of 28.2 (21.1–33.7) years and lived a median of 37.6 years shorter than their controls.

**Fig. 5:**
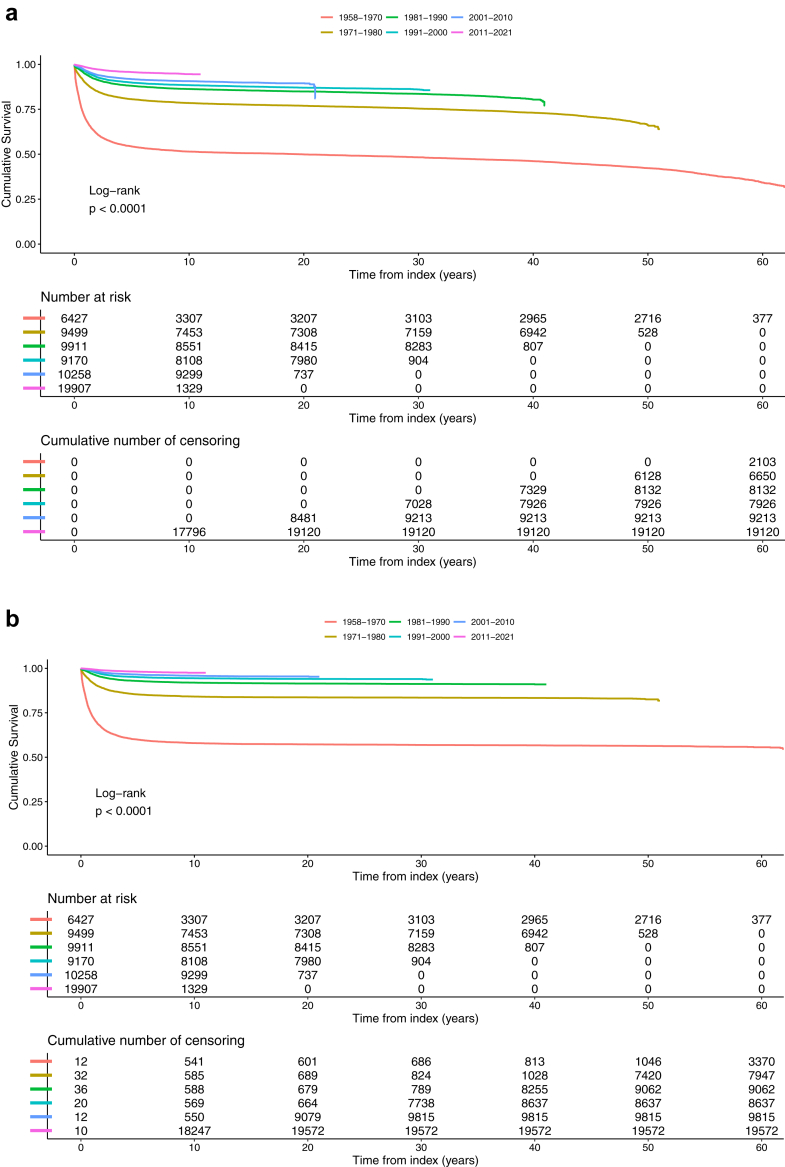

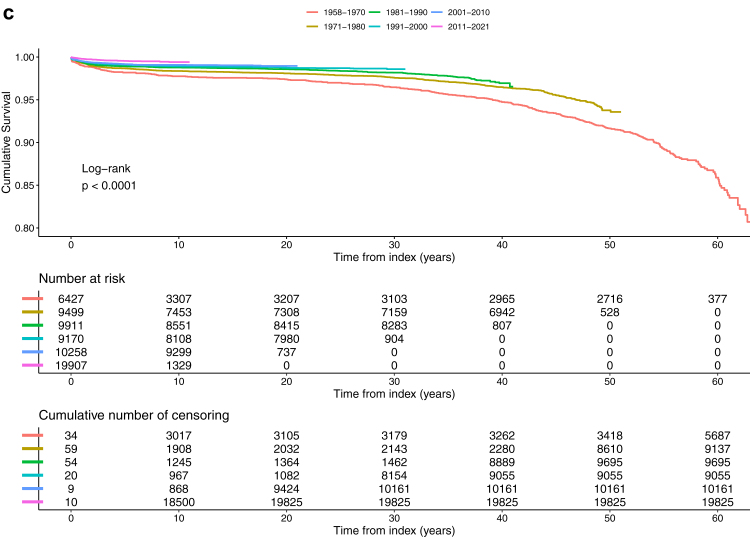
Survival trends over decades for individuals under 25 with cancer in Sweden between 1958 and 2021. Cumulative mortality for all-cause (a), cancer-specific (b), and cardiovascular (c) outcomes.

Cardiovascular (CV) mortality was also increased in CAYAs, (1.9% compared to 0.7% in controls) but at a higher age than in all-cause or cancer specific mortality, although 31.4 years younger than their controls. The five-years survivors were older at CV mortality and lived 5.0 years shorter than controls (Table 2, Fig. 5).

#### Mortality due to external causes

Regarding other deaths, 0.8% of CAYAs died due to injury, poisoning, suicide, or other external causes which was higher than in controls with CAYAs dying 1.5 year earlier.

All mortality causes are listed in Table 2 and illustrated in Fig. 5, and mortality for the 12 most common index diagnoses, age groups, and sex over the six decades are shown in Appendix p 7 and 8.

#### Mortality and socioeconomic factors

**Fig. 6:**
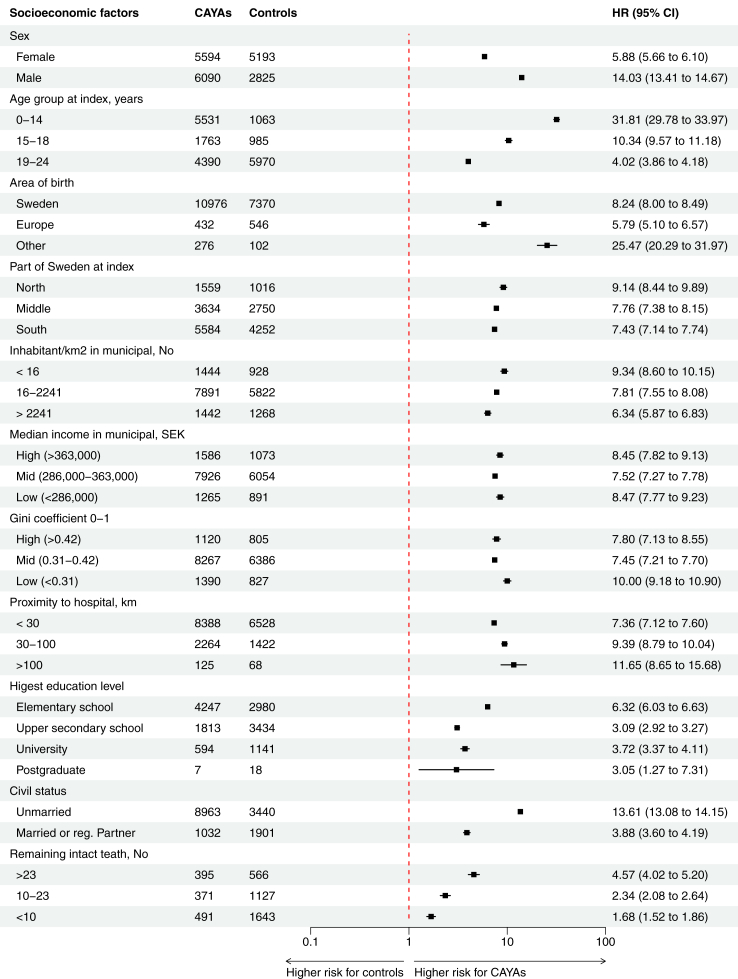
Hazard ratio (HR) plot (95% confidence interval (CI) and logarithmic scale) comparing demographic and socioeconomic factors between 11,684 deceased CAYAs and 8018 controls, with data from a total of 65,173 CAYAs and 312,935 matched controls. The factors that are considered high and low are based on the 10th and 90th percentiles. Adjusted data is presented in Supplement page 5 and 6. Abbreviations: CAYAs, children, adolescents, and young adults; No, number; Gini, coefficient ranging from 0 to 1, indicates income inequality within each municipality, with a higher value signifying greater inequality.

The mortality hazard for CAYAs, compared to controls, is illustrated in Fig. 6, divided into age, groups, demographic factors, and socioeconomic factors. After adjusting for demographic and socioeconomic factors male CAYAs had a 3.18-times higher risk for all-cause mortality compared to females (HR 95% CI 2.74–3.70, p < 0.0001) and 1.90-times increased risk among controls (HR 95% CI 1.72–2.10, p < 0.0001). (Supplement p 6) Most CAYAs and controls were born in Sweden (92.8% and 88.5%) and those CAYAs born outside Europe had a 2.19-times increased mortality risk (HR 95% CI 1.47–3.28, p < 0.0001). Most lived in the southern and middle parts of Sweden and only 12.3% in the north. Municipality median income, residence in north or south, living in an urban or a rural area or close to a hospital did not influence mortality in current study (Table 2 and Appendix p 5 and 6).

Among CAYAs, 15.5% had no more than primary school education, while 33.1% reached university and postgraduate levels, showing a difference between CAYAs and controls (Table 2). Of those who survived to the age of 30 years or more, 22% reached university level compared to 28% among the controls. Higher level of education, such as University, had a beneficial influence on mortality for both CAYAs (HR 0.53. 95% CI 0.43–0.66, p < 0.0001) and controls, (HR 0.43. 95% CI 0.38–0.49, p < 0.0001) (Appendix p 5 and 6).

In the CAYA group, 59.8% had not married, while 23.5% were married or had a registered partner. A higher number of married was seen in the control group (30.9%). In those that survived to an age of 30 years or more, 15.6% of the CAYAs were married compared to 20.6% controls. Being married was related with a 59% lower mortality risk for CAYAs, (HR 95% CI 0.36–0.48, p < 0.0001) and 62% for controls (HR 95% CI 0.35–0.42, p < 0.0001).

Disability pension was not connected with mortality, whereas having >180 days sick-leave was associated with 3.07-times increased mortality risk in CAYAs (HR 95% CI 2.67–3.53, p < 0.0001) and in controls 2.23-times higher risk (HR 95% CI 2.04–2.45, p < 0.0001). The number of intact teeth remaining was the same in both groups, with a higher number connected with increased mortality risk after adjusting for other socioeconomic factors (Table 2 and Appendix p 5 and 6).

## Discussion

In this comprehensive survey over the entire population of cancer patients diagnosed under the age of 25 in Sweden, our hypothesis that young cancer patients have increased morbidity and mortality risks across all forms of diseases was supported. The study further revealed that socioeconomic factors are related with an increased risk for mortality in CAYAs compared to matched controls.

The distribution of index malignancies was comparable to other international register studies, apart from the large number of young adults with cervical cancer, including high-grade squamous intraepithelial lesions, which has not been included in comprehensive reports from similar populations before. Human papillomavirus (HPV) infection is associated with cervical cancers, and it is known that survivors of cancer have a higher risk of HPV-associated cancers and CVD.^21^

Of the CAYAs that suffered from subsequent cancer, the children were at greatest risk. In a cancer population under 21 years of age, Sue et al.^3^ reported a 19.7% prevalence of different morbidities in children under 15 years compared to 13.6% in adolescents and young adults. Unfortunately, there are no reports on similar populations to enable comparisons with our results, so to what extent the high number of cervical cancers affects future health in this large population remains to be seen.

The proportion of CVD in the CAYAs was age-dependent and similar to that reported by Sue et al.^3^

The risk for other diseases was significantly higher in the CAYAs who showed much higher numbers of pulmonary, endocrine, and neurological diseases, than reported by Sue et al. Notably, they reported diseases of greater severity, which makes comparisons difficult since we included all diagnoses regardless of severity.

Cancer accounted for 54.7% of the total mortality. Fidler-Benaoudia et al.^13^ compared survivors between United States (US) and British childhood cancer survivor studies (CCSS and BCCSS) and found cancer specific mortalities of 57% and 75%, respectively. That study also showed that there were substantial differences in late all-cause mortality between the countries and suggested that US survivors may have received more intensive treatment and hence more late effects. While interpreting these and our results, it is essential to consider that variations in other healthcare factors may contribute to mortality differences between countries and continents, as they could influence the outcomes observed.

In a study by L. Wang et al.^22^ a 1.4-times higher CV mortality risk was observed among survivors aged 15–39 in the US compared to the general population, which is consistent with the present study comparing young adults with matched controls. In the present study, morbidity, and mortality from external causes was higher for CAYAs. This was also reported from the CCSS, and the same applied to the BCCSS cohort, though not to the same degree.^3^^,^^23^

### Mortality and socioeconomic factors

In Sweden, the proportion of children with a foreign background having a low-income standard is 23% compared to 4% for their Swedish counterparts. Having a background from outside, Europe had a negative link with mortality (no information on ethnicity). The most prosperous municipalities in Sweden i.e., those with good access to education, healthcare, and transportation, are found in the southern and middle parts. Other studies have revealed that a distance greater than 80 km to the nearest hospital may delay cancer diagnosis,^24^ increases mortality from leukaemia,^25^ and increase all-cause mortality.^26^^,^^27^ In current study neither greater distance to nearest hospital nor lower population density affected mortality for CAYAs, however this has yet to be analysed in different subgroups of index cancer such as leukaemia. Healthcare and socioeconomic factors influence the challenges regarding education and careers of cancer survivors. Previous studies have shown that cancer survivors often have their education and career plans disrupted, resulting in fewer receiving a college and university education.^28^ In current study, we observed that even though CAYAs passed primary school, subsequent education, even at higher ages, seemed to be hampered compared to controls, and that completing a university education was connected with lower mortality. This might indicate that good health is beneficial for higher studies (or vice versa). Civil status may be used as a surrogate for how well adapted the person became in adulthood. Living together in a joint household is as common as being married in Sweden, but this status is not registered, so half of all households in Sweden are officially single. In this study, CAYAs did not marry as often as their controls. They also had a valid difference in education and civil status when they were older than 30 years, having these factors were associated with mortality, indicating that the bias of high mortality in younger ages is not the dominant explanation of the findings.

Caries and number of teeth remaining is individual depending on age, nutrition, genetics, access to dental care, smoking and tobacco use, comorbidities, financial resources, education, and single living. The number of teeth needed for oral function is related to age, with a minimum of 24 teeth required under the age of 50.^29^ Both groups had the same number of teeth remaining, probably due to free dental care, but a high number of teeth was connected with higher mortality, an apparent contradiction that needs to be analysed more thoroughly.

Due to more illness among the CAYAs, they had more long-term sick leave over 180 days than the control group as Baecklund et al.^30^ have reported before. They found that female survivors were more likely to have more long-term sick leave later in life, which is a topic that needs more research in future Rebuc studies.

This study has strong internal validity because of the use of data from national high-quality patient registers with low dropout rates. However, register studies always have selection, confounding, measurement, and reporting biases. It is important to acknowledge the limitations arising from the use of data spanning different time periods, some of which may have incomplete overlap. Additionally, the study includes individuals who died before the establishment of certain registers.

This occurrence can be attributed to various factors such as incomplete data coverage, changes in registration criteria over time, potential data entry errors, and evolving healthcare practices. As a result, the analysis may not fully capture the morbidity patterns of individuals who passed away before the commencement of specific registers, as it may influence the generalizability and comprehensiveness of our conclusions. We must also acknowledge the built-in selection bias in the HR analyses.^31^ In addition, it's essential to recognize the limitation regarding treatment information, which was not present in the current registries and not further analysed in this study. Thus, while our study provides valuable insights, it's crucial to acknowledge that the lack of treatment data could potentially impact the comprehensiveness of our findings and limit the depth of our analysis.

The assessment of changes in the management of cancer in CAYAs over time in this comprehensive study may be considered somewhat broad to drawing any definitive conclusions. However, it serves as a foundational framework, and construction of a large database, for initiating more in-depth studies to gain a deeper understanding of the subject and to draw conclusions of.

This study includes data on five-year survivors who had subsequent cancers and examines five-years survivals in mortality, hospital stay, marriage and education, making it comparable to other studies of survivals. This is the first extensive study of young cancer patients in Sweden, and it might be relevant to recognize diseases that occur within five years of a cancer diagnosis when examining the demographic and socioeconomic factors, because many of the diseases within five years after cancer diagnosis may act as a risk factor for other diseases or socioeconomic outcome throughout life.

Increased morbidity and higher risk for other disease over time in the CAYA group probably reduces quality of life, but the significance of this has yet to be shown. The long-term consequences and cost-effectiveness of modern forms of cancer management also need investigating. Future research should also focus on developing tailored interventions to address the holistic well-being and social integration of young cancer survivors. This involves conducting longitudinal studies to understand survivors evolving needs, developing multidisciplinary interventions, implementing peer support programs, ensuring access to resources, and engaging with communities to foster support networks. By prioritizing these efforts, we can enhance the overall quality of life for cancer survivors and promote their long-term health and social outcomes.

### Conclusion

This study shows that young cancer patients not only face a higher risk for developing a new malignancy but also suffer increased cardiovascular and other morbidities. The higher mortality risk before middle age underscores the severity of the health challenges they face. Additionally, socio-economic factors like; being born outside of Europe, having no higher education, or living without spouse increased the likelihood of death in CAYAs compared to controls.

## Contributors

All authors contributed to the study's conception and design. The entire team collaborated on translating ICD codes and all other data collection was performed by LH, MSt, JA, BE and LE. RK data managed and established the database. MSi, LH and PM did the statistical analyses. The first drafts of the manuscript were written by LH, PM and EH and all authors commented on subsequent versions of the manuscript. All authors have read and approved the final manuscript.

## Data sharing statement

The datasets generated and analysed in this study are not available to the public due to Swedish laws and regulations. However, they can be made accessible with the support of investigators and upon reasonable request, in accordance with Swedish healthcare secrecy legislation.

## Declaration of interests

EH is Co-Founder and board member of MedTech-company TrueDose AB, producing at-home blood sampling kits. EH has received speaker's and consultancy fees from Bristol-Myers Squibb, Pfizer, and Amgen. KRW reports unrelated speaker's and consultancy fees from Roche, Pfizer, Organon, Ibsa, Merck and Ferring pharmaceuticals and unrelated grants from Novo Nordisk and Ferring. LE is a board member of the Swedish Intensive Care Register. JA has received lecture fees from Boehringer Ingelheim, Astra Zeneca, MSD, Bayer and Novartis (modest) and advisory board reimbursement from Astra Zeneca and Bayer (modest). He is chair of the board of SWEDEHEART ACS register and the SWEDEHEART Register Research Council and member of the SWEDEHEART steering committee. JM is unpaid member of the board of the Swedish Multiple Sclerosis Society. ME reports unrelated consultation fees from Bayer, Thea Pharma and Novartis. LH reports unrelated modest consultation fees from Astellas, Bayer, and Orion Pharma. All remaining authors declare that they have no conflict of interest.

## References

[1] NationalBoardofHealthandWelfare (2023). Statistics and data. https://www.socialstyrelsen.se/en/statistics-and-data/.

[2] Winther J.F., Kenborg L., Byrne J. (2015). Childhood cancer survivor cohorts in Europe. Acta Oncol.

[3] Suh E., Stratton K.L., Leisenring W.M. (2020). Late mortality and chronic health conditions in long-term survivors of early-adolescent and young adult cancers: a retrospective cohort analysis from the childhood cancer survivor study. Lancet Oncol.

[4] de Fine Licht S., Rugbjerg K., Gudmundsdottir T. (2017). Long-term inpatient disease burden in the adult life after childhood cancer in scandinavia (ALiCCS) study: a cohort study of 21,297 childhood cancer survivors. PLoS Med.

[5] Gudmundsdottir T., Winther J.F., de Fine Licht S. (2015). Cardiovascular disease in adult life after childhood cancer in scandinavia: a population-based cohort study of 32,308 one-year survivors. Int J Cancer.

[6] Delaney A., Howell C.R., Krull K.R. (2021). Progression of frailty in survivors of childhood cancer: a St. Jude lifetime cohort report. J Natl Cancer Inst.

[7] Teepen J.C., van Leeuwen F.E., Tissing W.J. (2017). Long-term risk of subsequent malignant neoplasms after treatment of childhood cancer in the DCOG LATER study cohort: role of chemotherapy. J Clin Oncol.

[8] de Baat E.C., Feijen E.A.M., Reulen R.C. (2023). Risk factors for heart failure among pan-European childhood cancer survivors: a PanCareSurFup and ProCardio cohort and nested case-control study. J Clin Oncol.

[9] Dixon S.B., Liu Q., Chow E.J. (2023). Specific causes of excess late mortality and association with modifiable risk factors among survivors of childhood cancer: a report from the childhood cancer survivor study cohort. Lancet.

[10] Meijers W.C., de Boer R.A. (2019). Common risk factors for heart failure and cancer. Cardiovasc Res.

[11] Lipshultz S.E., Franco V.I., Miller T.L., Colan S.D., Sallan S.E. (2015). Cardiovascular disease in adult survivors of childhood cancer. Annu Rev Med.

[12] Herrera D., Sanz M., Shapira L. (2023). Association between periodontal diseases and cardiovascular diseases, diabetes and respiratory diseases: consensus report of the joint workshop by the European federation of periodontology (EFP) and the European arm of the world organization of family doctors (WONCA Europe). J Clin Periodontol.

[13] Fidler-Benaoudia M.M., Oeffinger K.C., Yasui Y. (2021). A comparison of late mortality among survivors of childhood cancer in the United States and United Kingdom. J Natl Cancer Inst.

[14] Byrne J., Schmidtmann I., Rashid H. (2022). Impact of era of diagnosis on cause-specific late mortality among 77 423 five-year European survivors of childhood and adolescent cancer: the PanCareSurFup consortium. Int J Cancer.

[15] Boman K.K., Lindblad F., Hjern A. (2010). Long-term outcomes of childhood cancer survivors in Sweden: a population-based study of education, employment, and income. Cancer.

[16] Di Giuseppe G., Pagalan L., Jetha A., Pechlivanoglou P., Pole J.D. (2023). Financial toxicity among adolescent and young adult cancer survivors: a systematic review of educational attainment, employment, and income. Crit Rev Oncol Hematol.

[17] Hjelmstedt S., Lindahl Norberg A., Montgomery S., Hed Myrberg I., Hovén E. (2017). Sick leave among parents of children with cancer - a national cohort study. Acta Oncol.

[18] Ritter J., Allen S., Cohen P.D. (2023). Financial hardship in families of children or adolescents with cancer: a systematic literature review. Lancet Oncol.

[19] Ruiz S., Hudson M.M., Ehrhardt M.J., Maki J., Ackermann N., Waters E.A. (2023). Childhood cancer survivors, financial toxicity, and the need for multilevel interventions. Pediatrics.

[20] Schwartz L.F., Dhaduk R., Howell C.R. (2023). The association of neighborhood characteristics and frailty in childhood cancer survivors: a report from the St. Jude lifetime cohort study. Cancer Epidemiol Biomarkers Prev.

[21] Liang X., Chou O.H.I., Cheung B.M.Y. (2023). The effects of human papillomavirus infection and vaccination on cardiovascular diseases, NHANES 2003-2016. Am J Med.

[22] Wang L., Wang F., Chen L., Geng Y., Yu S., Chen Z. (2021). Long-term cardiovascular disease mortality among 160 834 5-year survivors of adolescent and young adult cancer: an American population-based cohort study. Eur Heart J.

[23] Fidler M.M., Reulen R.C., Henson K. (2017). Population-based long-term cardiac-specific mortality among 34 489 five-year survivors of childhood cancer in great britain. Circulation.

[24] Johnson K.J., Wang X., Barnes J.M., Delavar A. (2021). Associations between geographic residence and US adolescent and young adult cancer stage and survival. Cancer.

[25] Rotz S.J., Wei W., Thomas S.M., Hanna R. (2020). Distance to treatment center is associated with survival in children and young adults with acute lymphoblastic leukemia. Cancer.

[26] Nicholl J., West J., Goodacre S., Turner J. (2007). The relationship between distance to hospital and patient mortality in emergencies: an observational study. Emerg Med J.

[27] Wei L., Lang C.C., Sullivan F.M. (2008). Impact on mortality following first acute myocardial infarction of distance between home and hospital: cohort study. Heart.

[28] Devine K.A., Christen S., Mulder R.L. (2022). Recommendations for the surveillance of education and employment outcomes in survivors of childhood, adolescent, and young adult cancer: a report from the international late effects of childhood cancer guideline harmonization group. Cancer.

[29] Edman K., Öhrn K., Nordström B., Holmlund A. (2016). Prevalence of dental caries and influencing factors, time trends over a 30-year period in an adult population. Epidemiological studies between 1983 and 2013 in the county of Dalarna, Sweden. Acta Odontol Scand.

[30] Baecklund F., Alexanderson K.A.E., Mittendorfer-Rutz E., Chen L. (2022). Sickness absence and disability pension trajectories in childhood cancer survivors and references- a Swedish prospective cohort study. PLoS One.

[31] Hernan M.A. (2010). The hazards of hazard ratios. Epidemiology.

