# Supplementary material for: Morbidity and mortality among children, adolescents, and young adults with cancer over six decades: a Swedish population-based cohort study (the Rebuc study)
Source: Lancet Reg Health Eur. 2024 May 15;42:100925. doi: 10.1016/j.lanepe.2024.100925 (PMC11126812; doi:10.1016/j.lanepe.2024.100925)
Supplement: Supplementary Appendix [file mmc1.pdf]

Supplementary appendix

	Page
Municipalities and healthcare regions in Sweden	1
**Supplementary Figures**	
Figure 1. Flowchart of the study population.	2
Figure 2. National registers over time	2
Figure 3. Cumulative mortality for 12 index diagnoses	7
Figure 4. All-cause mortality in males and females and in the three age groups	8
**Supplementary Tables**	
Tabel 1. The National Board of Health and Welfare's Registers	3
Table 2. ICD 10 codes and the translation process from ICD 7-9.	4
Tabel 3. Time, age, sex, and socioeconomic factors/covariates impacts on mortality. Unadjusted	5
Table 4. Sex and Socioeconomic impacts on all-cause mortality. Adjusted	6

Municipalities

Sweden has a total population of just over 10 million inhabitants and is divided into three main parts: northern (Norrland, 11·4%), middle (Svealand, 40·8%), and southern Sweden (Gotaland, 47·8%). In 2021 there were 290 municipalities each with local differences in median income. The Gini coefficient, which ranges from 0-1, indicates income inequality within each municipality, with a higher value signifying greater inequality.[1] Municipalities vary between 2395 and 978770 inhabitants and southern Sweden is much more densely populated than the northern part of the country.

Health-care system

There are 21 healthcare regions, each with an average of 500 000 inhabitants.
The healthcare regions have great autonomy and are responsible for the healthcare organisation within a framework set by the state. There are seven university hospitals, 23 regional hospitals, and 38 general hospitals. All hospitals provide an emergency service, but special healthcare services are restricted to the first two hospital categories only. Healthcare in Sweden is financed by taxes (85%) and patient fees (14%), and only 1% is covered by a voluntary health insurance scheme. Dental care is free up to 23 years of age and then partly covered by public health care.[2]

All healthcare regions contribute to several national healthcare quality registers, aiming to develop and ensure the quality of care, produce data for statistics and research, and make it possible to compare and improve healthcare at national and regional levels. These registers contain sensitive information, but Sweden has rigorous data privacy regulations to protect the individual's integrity. Access to register data is strictly controlled and granted for research purposes after ethical and legal considerations. The National Board of Health and Welfare has a register containing a wide range of healthcare-related data, including diagnoses, treatments, prescriptions, hospital admissions, births and deaths, and cancer (the Cancer Register that, since 1958, has comprehensive data on all forms of cancer). These registers cover the entire of Sweden, providing a complete and accurate representation of its population's health with standard coding and reporting practices. Consequently, these registers are reliable sources of data for research and statistics.

1.Hasell J. Measuring inequality: What is the Gini coefficient? https://ourworldindata.org/what-is-the-gini-coefficient2023

2.National Board of Health and Welfare. Statistics and Data 2023 [Available from: https://www.socialstyrelsen.se/en/statistics-and-data/.

Figure 1. Flowchart of the study population.

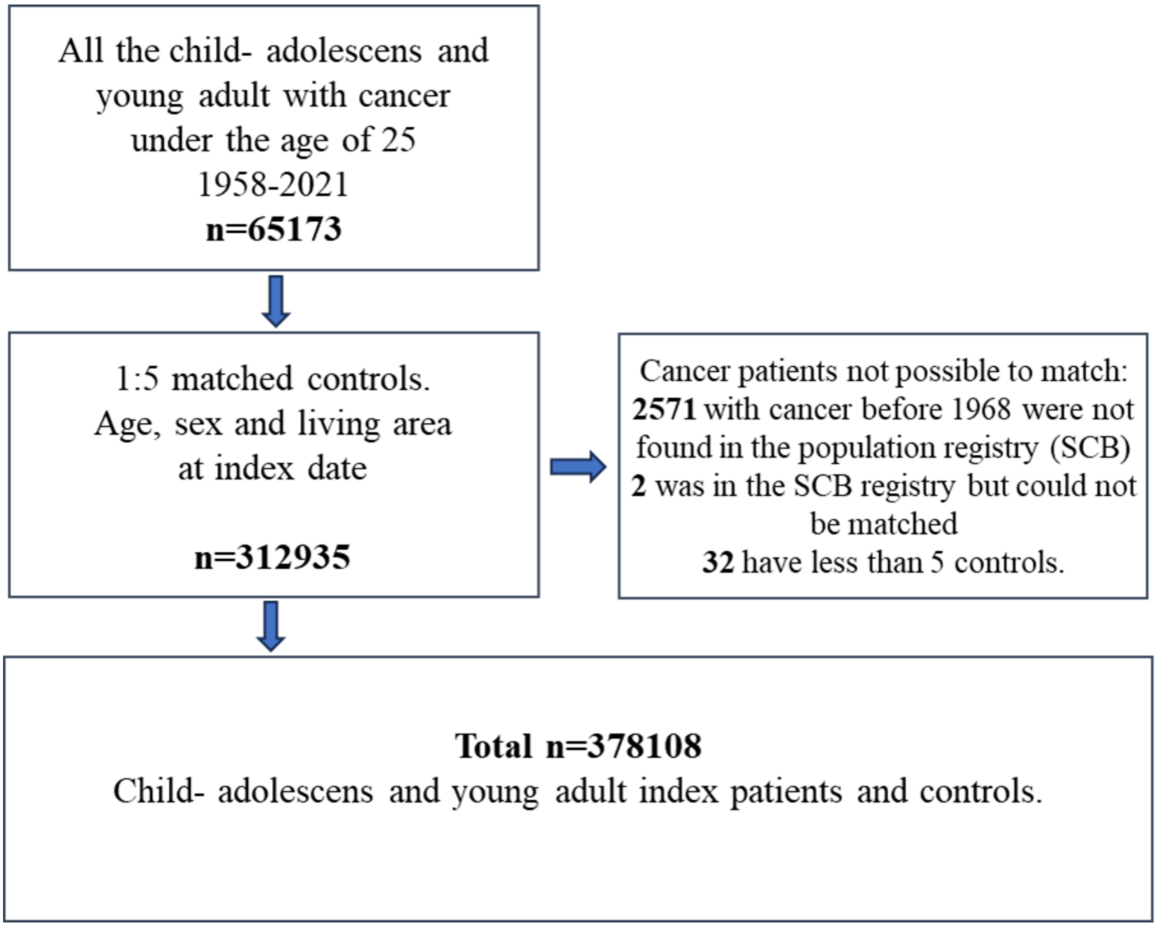

Figure2. Swedish National registers used in this study and their timeframe.

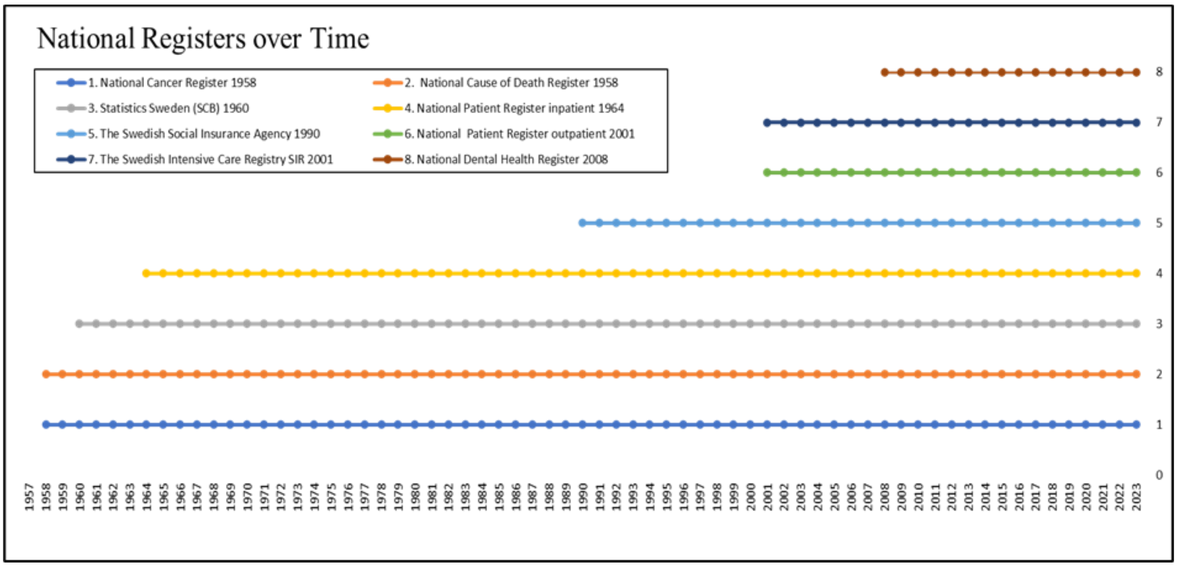

Table 1. Swedish National Quality registers used to obtain information data on cancer patients and their controls.

Register[1]	Information	Information retrieved
National Cancer Register 1958-2021	Started in 1958 and includes data on histological type, site, date of diagnosis, date and cause of death, with a coverage rate of 96%. All tumours in the register are coded and registered according to ICD-7 and ICD10.[2,3]	Index cancer diagnosis and date
The Cause of Death Register 1952-2021	The Swedish Cause of Death Register is a high quality virtually complete register of all deaths in Sweden since 1952.[4]	Cause of death and date
Statistics Sweden 1960-2021	Statistics Sweden (SCB) is responsible for official statistics and for other government statistics since 1960.<br>SCB also provide a Longitudinal integrated database for health insurance and labour market studies (LISA)	Matched controls1:5<br>Residence at index diagnosis (municipality number at index diagnosis), civil status (latest registered), sex (assigned at birth) and education levels (latest registered).
The Swedish Social Insurance Agency 1990-2021	Conducts supporting research in the field of social insurance. Investigates and decides on the right to compensation from social insurance. It includes, amongst other things, compensation for sickness benefit.	Days sick-leave and disability pension
National Patient Register. 1964-2021	Captures data on hospitalised patients (inpatients) and those who have received medical care from healthcare providers without hospital admission (outpatients) for diagnosis, treatment, or follow-up. The inpatient register has been operational since 1964, while the outpatient register was started in 2001. These registers are essential to healthcare information infrastructure, monitoring, and improving healthcare services nationwide.[5]	Diagnosis after index date and days in hospital care. Additional diagnoses were collected from the outpatient register.
National Dental Health Register 2008-2021	The Swedish Dental Health Register contains individual data on dental health care to the whole adult population of Sweden[6]	Number of intact teeth.
The Swedish Intensive Care Register SIR 2001-2021	Intensive care in the register is defined as advanced monitoring, diagnosis, or treatment of imminent or manifest failure of vital bodily functions.	Time on intensive care unit since 2008

References:
1.Ludvigsson JF, Almqvist C, Bonamy AK, Ljung R, Michaelsson K, Neovius M, et al. Registers of the Swedish total population and their use in medical research. Eur J Epidemiol. 2016;31(2):125-36.
2.Socialstyrelsen. Swedish National Cancer Register. Available from: https://www.socialstyrelsen.se/en/statistics-and-data/registers/register-information/swedish-cancer-register/.
3. Barlow L, Westergren K, Holmberg L, Talback M. The completeness of the Swedish Cancer Register: a sample survey for year 1998. Acta Oncol. 2009;48(1):27-33.
4.Brooke HL, Talback M, Hornblad J, Johansson LA, Ludvigsson JF, Druid H, et al. The Swedish cause of death register. Eur J Epidemiol. 2017;32(9):765-73.
5.Ludvigsson JF, Andersson E, Ekbom A, Feychting M, Kim JL, Reuterwall C, et al. External review and validation of the Swedish national inpatient register. BMC Public Health. 2011;11:450.
6.Ljung R, Lundgren F, Appelquist M, Cederlund A. The Swedish dental health register - validation study of remaining and intact teeth. BMC Oral Health. 2019;19(1):116.

**Table 2. ICD 7-9 codes from 1958-1997 were translated into ICD-10 codes.**	
**Malignancy**	**ICD-10**
Lip, oral cavity, and pharynx	C00-14
Oesophagus	C15
Stomach and small intestine	C16-17
Colon	C18-21
Liver and gallbladder	C22-24
Pancreas	C25
Other gastrointestinal	C26
Airways, mouth, larynx, trachea, and lung	C30-34
Bone, connective, and soft tissue	C40-41
Skin cancer	C43-44
Breast	C50
Female reproductive	C51-52, C54-58
Cervix	C53
Prostate	C61
Testis	C62
Other genitourinary	C63
Kidney and bladder	C64-68
CNS	C69-72
Thyroid and other endocrine glands	C73-75
Lymphoma	C81-86
Leukaemia	C91-95
Benign meningioma	D32
All other specified neoplasms	C37-39, C45-49, C60, C88, C90
**Cardiovascular diseases**	**I00-I99**
Coronary artery diseases	I20-I25
Pulmonary embolism	I26
Myo-, endo- and pericarditis	I32, I33, I40, I41
Arrhythmias	I44-I49
Heart failure and cardiomyopathy	I50, I42, I43
Valvular diseases	I34-I37
Cerebrovascular	I60-I69
Hypertension	I10
**Pulmonary Diseases**	**J00-J99**
Chronic lower respiratory disease	J44
Interstitial lung disease, including fibrosis	J84
**Other health-related cause**	**A00-Y98**
Infectious and parasitic	A00-B99
Blood and blood-forming organs	D50-D89
Thyroid and other endocrine glands	E00-E90
Mental illness	F00-F99
Neurological	G00-G99
Eye	H00-H59
Ear	H60-H95
Gastrointestinal	K00-K93
Skin	L00-L99
Musculoskeletal	M00-M99
Kidney and genitourinary	N00-N99
Injury, poisoning, and other of external causes	V01-Y98

The translation to ICD-10 was based on the recommendations for mapping from ICD-9 to ICD-10 from Socialstyrelsen[1], the electronic supplementary material for converting between ICD-8 and ICD-10 by Pedersen et al.[2], and manual matching by medical professionals. ICD 7-9 codes for translation are available at:

1. Socialstyrelsen. National Board of Health and Welfare. Historical ICD coding available from: https://www.socialstyrelsen.se/statistik-och-data/klassifikationer-och-koder/icd-10/
2. Pedersen MK, Eriksson R, Reguant R, Collin C, Pedersen HK, Sorup FKH, et al. A unidirectional mapping of ICD-8 to ICD-10 codes, for harmonised longitudinal analysis of diseases. Eur J Epidemiol. 2023.

Tabel 3. Time, age, sex and socioeconomic factors and covariates and all-cause mortality (univariable) for the 11 684 CAYAs and 8018 Controls who died.

	CAYAs				Controls			
	HR	95% CI		p-value	HR	95% CI		p-value
**Sex at index**								
Female	ref				ref			
Male	3·50	3·38	3·63	<0·0001	1·46	1·40	1·53	<0·0001
**Decade at index**								
1958-1970	ref				ref			
1971-1980	0·41	0·39	0·43	<0·0001	0·86	0·81	0·92	<0·0001
1981-1990	0·26	0·24	0·27	<0·0001	0·67	0·63	0·75	<0·0001
1991-2000	0·20	0·19	0·21	<0·0001	0·57	0·51	0·64	<0·0001
2001-2010	0·16	0·15	0·17	<0·0001	0·57	0·51	0·67	<0·0001
2011-2021	0·08	0·07	0·09	<0·0001	0·60	0·50	0·71	<0·0001
**Age at index· years**	0·93	0·93	0·93	<0·0001	1·06	1·05	1·06	<0·0001
<1-14	ref				ref			
15-18	0·63	0·60	0·67	<0·0001	1·91	1·75	2·08	<0·0001
19-24	0·24	0·23	0·25	<0·0001	2·34	2·20	2·50	<0·0001
**Area of birth**								
Sweden	ref				ref			
Europe	0·81	0·74	0·90	<0·0001	1·03	0·95	1·13	0·49
Other	0·90	0·80	1·02	0·088	0·74	0·61	0·90	0·0029
**Part of Sweden at index**								
South	ref				ref			
Middle	1·09	1·04	1·13	<0·0001	1·07	1·02	1·12	0·0048
North	1·28	1·21	1·35	<0·0001	1·09	1·02	1·17	0·017
**Inhabitant/km2 in municipal. no**	1·00	1·00	1·00	<0·0001	1·00	1·00	1·00	<0·0001
≥ 2241	ref				ref			
16-2241	0·95	0·90	1·01	0·14	0·86	0·82	0·92	<0·0001
<16	1·35	1·25	1·45	<0·0001	0·97	0·89	1·06	0·49
**Median income in municipal. SEK**	1·00	1·00	1·00	<0·0001	1·00	1·00	1·00	0·030
> 363 000	ref				ref			
286 000-363 000	0·94	0·89	0·99	0·018	0·99	0·93	1·06	0·80
< 286 000	1·20	1·11	1·29	<0·0001	1·06	0·97	1·16	0·17
**Gini coefficient 0-1**	0·06	0·04	0·09	<0·0001	1·40	0·84	2·35	0·20
<0·31	ref				ref			
0·31-0·42	0·71	0·67	0·75	<0·0001	1·002	0·93	1·08	0·95
>0·42	0·74	0·68	0·80	<0·0001	1·02	0·93	1·12	0·67
**Proximity to hospital. km**	1·00	1·00	1·01	<0·0001	1·00	1·00	1·00	0·45
<30	ref				ref			
30-100	1·24	1·19	1·30	<0·0001	0·94	0·89	1·00	0·040
> 100	1·90	1·59	2·26	<0·0001	1·07	0·84	1·36	0·572
**Highest education level**								
Elementary school	ref				ref			
Upper secondary school	0·15	0·14	0·16	<0·0001	0·40	0·38	0·42	<0·0001
University	0·06	0·06	0·07	<0·0001	0·17	0·15	0·18	<0·0001
Postgraduate	0·04	0·02	0·08	<0·0001	0·09	0·06	0·14	<0·0001
**Civil status**								
Unmarried	ref				ref			
Married or reg Partner	0·19	0·18	0·20	<0·0001	0·36	0·34	0·38	<0·0001
**Remaining intact teeth. no**	1·00	1·00	1·01	<0·0001	0,99	0·98	0·99	0·00021
<10	1·23	1·07	1·41	0·0040	1·44	1·33	1·55	<0·0001
10-23	ref				ref			<0·0001
≥24	2·20	1·90	2·54	<0·0001	1·51	1·36	1·67	<0·0001
**Sick-leave and disability pension days**								
Sick-leave >180	0·54	0·51	0·57	<0·0001	1·71	1·63	1·79	<0·0001
Total sick-leave + disability pension	1·00	1·00	1·00	<0·0001	1·00	1·00	1·00	<0·0001

Number of CAYAs and Controls in each demographic and socioeconomic analysis are listed in Figure 6. Abbreviations: SEK= Swedish crowns. no=numbers. CAYA= child, adolescent, and young adult. km=kilometres. HR= hazard ratio. CI=confidence interval.

Table 4. Sex and Socioeconomic factors and all-cause mortality. Adjusted for 12 factors (multivariable) for the 11 684 CAYAs and 8018 Controls who died.

	CAYAs				Controls			
	HR	95% CI		p-value	HR	95% CI		p-value
**Sex at index**								
Female	ref				ref			
Male	3.18	2.74	3.70	<0·0001	1·90	1·72	2·10	<0·0001
**Age at index**	1·08	1·06	1·09	<0·0001	1·10	1·09	1·11	<0·0001
**Area of birth**								
Sweden	ref				ref			
Europe	1.32	0.95	1.83	0.098	0·83	0·66	1·05	0·12
Other	2.19	1.47	3.28	<0·0001	1·70	1·18	2·45	0·0042
**Part of Sweden at index date**								
South	ref				ref			
Middle	1·17	0·99	1·37	0·062	1·04	0·93	1·16	0·54
North	0·93	0·72	1·21	0·60	1·05	0·90	1·24	0·53
**Inhabitant/km2 in municipal. no**								
≥ 2241	ref				ref			
16-2241	1·08	0·82	1·33	0·57	0·87	0·73	1·02	0·092
<16	0·95	0·63	1·44	0·82	1·04	0·80	1·34	0·77
**Median income in municipal. SEK**								
> 363 000	ref				ref			
286 000-363 000	0.99	0·73	1·33	0·94	1·04	0·84	1·29	0·74
< 286 000	1·08	0·74	1·33	0·67	0·98	0·76	1·28	0·92
**Gini coefficient 0-1**								
<0·31	ref				ref			
0·31-0·42	0·81	0·62	1·04	0·10	1·33	1·11	1·58	0·0016
>0·42	0·74	0·47	1·16	0·19	1·28	0·95	1·74	0·10
**Proximity to hospital. km**								
<30	ref				ref			
30-100	0·89	0·72	1·09	0·25	0·96	0·84	1·09	0·55
> 100	0·66	0·24	1·83	0·43	1·02	0·62	1·66	0·93
**Highest education level**								
Elementary school	ref				ref			
Upper secondary school	0·89	0·74	1·07	0·21	0·75	0·68	0·84	<0·0001
University	0·53	0·43	0·66	<0·0001	0·43	0·38	0·49	<0·0001
Postgraduate	0·43	0·14	1·34	0·14	0·34	0·18	0·63	0·00070
**Civil status**								
Unmarried	ref				ref			
Married or reg· Partner	0·41	0·36	0·48	<0·0001	0·38	0·35	0·42	<0·0001
**Remaining intact teeth. no**								
<10	0·99	0·83	1·20	0·96	1·01	0·92	1·13	0·74
10-23	ref				ref			
≥24	2·05	1·73	2·43	<0·0001	1·54	1·36	1·75	<0·0001
**Sick-leave and disability pension days**								
Sick-leave >180	3·07	2·67	3·53	<0·0001	2·23	2·04	2·45	<0·0001

Number of CAYAs and Controls in each demographic and socioeconomic analysis are listed in Figure 6. Abbreviations: SEK= Swedish crown. no=numbers CAYA= child, adolescent, and young adult. km=kilometres. HR= hazard ratio. CI=confidence interval.

Figure 3 Cumulative all-cause, cancer specific and cardiovascular mortality for 12 index diagnosis.

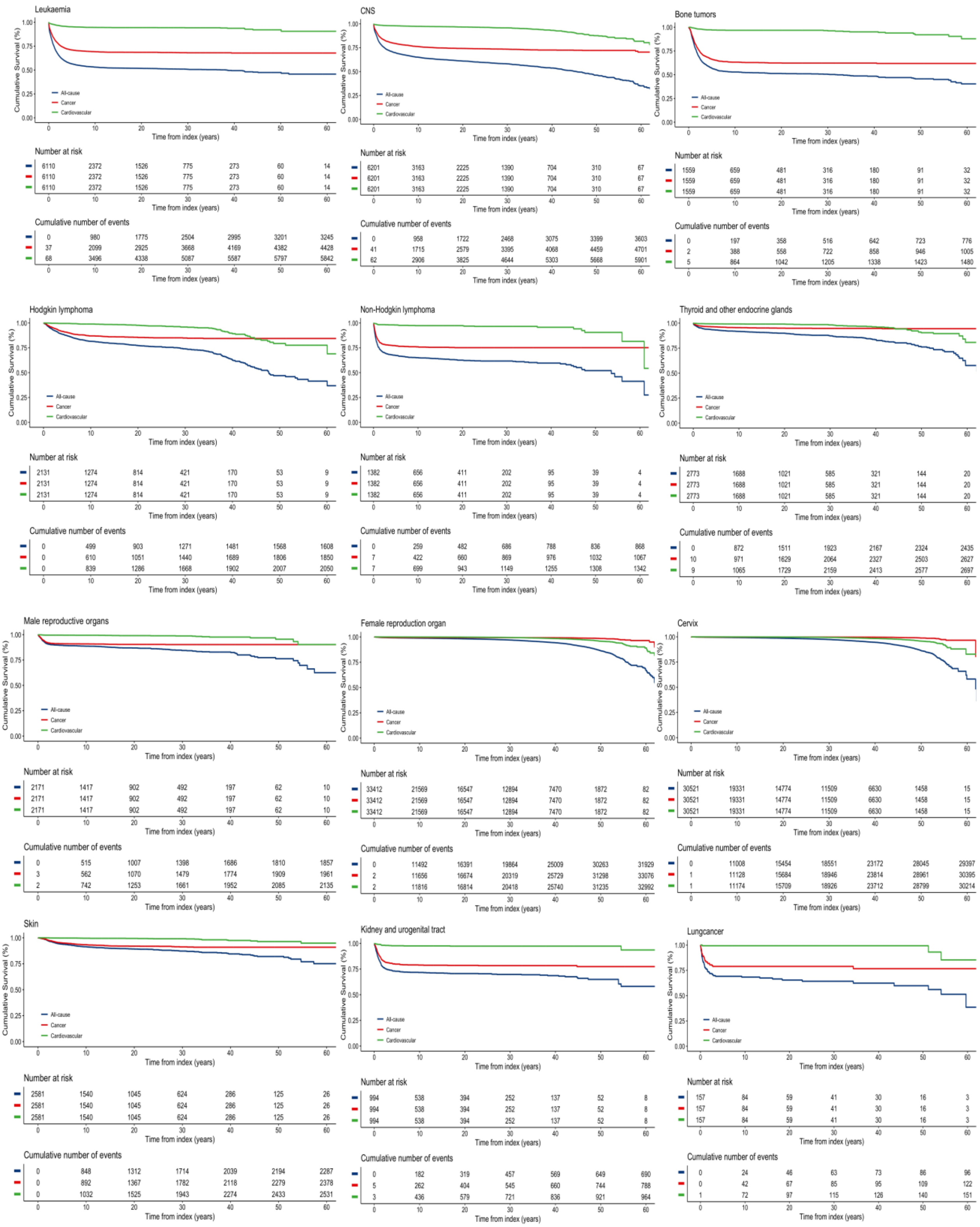

Figure 4. All-cause mortality in males and females (top) and in age groups (<1-14, 15-18, and 19-24) (below)

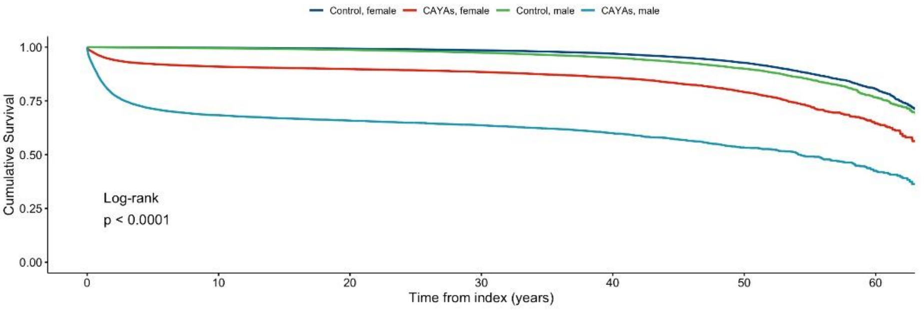

Number at risk

	0	10	20	30	40	50	60
	232644	158202	119557	89355	52138	15719	1285
	47673	29155	21653	15970	9034	2579	242
	80291	61536	45602	30214	17256	6421	733
	17499	8892	5994	3479	1680	665	135

Time from index (years)

Cumulative number of censoring

	0	10	20	30	40	50	60
	0	73847	111877	141320	177452	212515	226259
	0	14498	21683	27086	33656	39698	41857
	0	18372	33914	48803	61213	71428	76787
	0	3298	5918	8275	9932	10821	11287

Time from index (years)

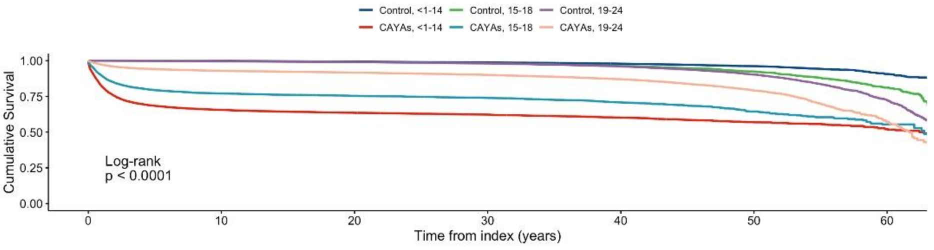

Number at risk

	0	10	20	30	40	50	60
	69189	54482	41249	27693	16397	6158	825
	15293	7635	5288	3127	1583	660	160
	31071	24854	18945	13795	8304	2872	437
	6638	4058	2970	2105	1223	418	84
	212675	140402	104965	78081	44693	13110	756
	43241	26354	19389	14217	7908	2166	133

Time from index (years)

Cumulative number of censoring

	0	10	20	30	40	50	60
	0	14581	27606	40952	52059	62102	67317
	0	2590	4725	6800	8268	9136	9608
	0	6091	11879	16877	22176	27392	29686
	0	1112	2123	2941	3754	4491	4797
	0	71547	106306	132294	164430	194449	206043
	0	14094	20753	25620	31566	36892	38739

Time from index (years)
